# Hydrogen Atom Transfer-Based C(sp^3^)–H Bond Oxygenation of Lactams and Cycloalkenes: The Influence of Ring Size on Reactivity and Site Selectivity

**DOI:** 10.1021/acs.joc.5c00092

**Published:** 2025-04-07

**Authors:** Sergio Sisti, Fabio Ioele, Filippo Scarchilli, Simona Laparelli, Marco Galeotti, Omid Hosseinzadeh, Zhehan Jia, Gino A. DiLabio, Michela Salamone, Massimo Bietti

**Affiliations:** † Dipartimento di Scienze e Tecnologie Chimiche, Università “Tor Vergata”, Via della Ricerca Scientifica, 1 I-00133 Rome, Italy; ‡ QBIS Research Group, Institut de Química Computacional i Catàlisi (IQCC) and Departament de Química, Universitat de Girona, Campus Montilivi, Girona E-17071, Catalonia, Spain; § Department of Chemistry, The University of British Columbia, 3247 University Way, Kelowna, British Columbia V1V 1V7, Canada

## Abstract

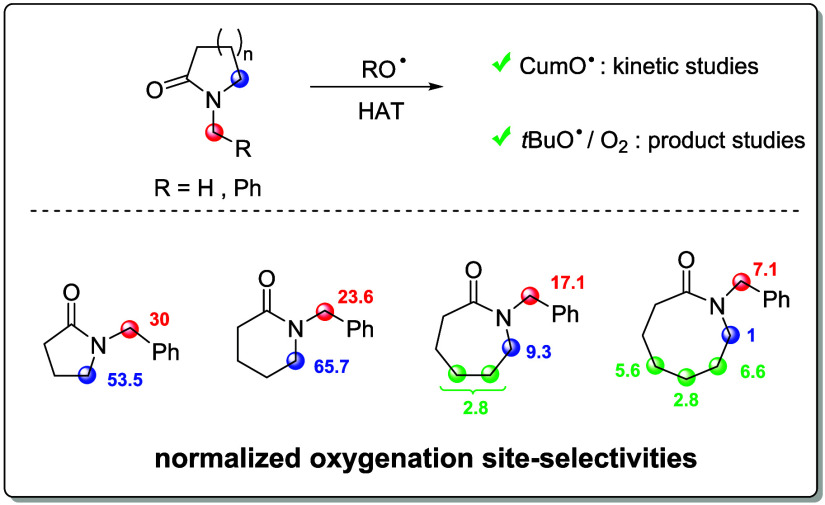

Kinetic and product studies on the reactions of the cumyloxyl (CumO^•^) and *tert*-butoxyl (*t*BuO^•^) radicals with secondary and tertiary *N*-methyl and *N*-benzyl lactams and with cycloalkenes, accompanied by BDE calculations of the substrate C–H bonds involved in these reactions, are reported. Within the lactams, the rate constants for HAT (*k*_H_) from the C–H bonds to CumO^•^ decrease by a factor ∼4 going from the 5- and 6-membered derivatives to the 8-membered ones. Product distributions obtained through oxygenation initiated by *t*BuO^•^ show that HAT preferentially occurs from the most electron-rich α-C–H bonds, with site selectivity that, within the *N*-methyl and *N*-benzyl series, progressively shifts from the endocyclic to the exocyclic α-C–H bonds with increasing ring size, indicative of deactivation of the former bonds that, for the 8-membered derivatives, translates into competitive oxygenation at different sites. Similar trends have been observed for the corresponding reactions of the cycloalkenes, with *k*_H_ values that decrease by a factor of ∼4 together with site selectivity for HAT from the activated allylic C–H bonds, going from cyclopentene to cyclooctene. It is proposed that the greater flexibility of the medium-sized rings decreases the extent of hyperconjugative overlap between the α-C–H bonds and the amide or C=C π-systems, increasing the kinetic barrier for HAT from these sites, with decreases in reactivity that approach factors of 83 and 18, for the endocyclic α-C–H bonds of tertiary *N*-methyl lactams and the allylic C–H bonds of cycloalkenes, respectively.

## Introduction

Nitrogen-containing heterocycles are common fragments in active pharmaceuticals and natural products and represent important building blocks and attractive synthetic targets in the design and discovery of bioactive materials.^1^ Among these widespread and diverse heterocyclic compounds, lactams represent an important class.^2a,2b^ Lactam-containing molecules have therapeutic applications in different fields including oncology, neurology, vascular, and infectious diseases, with γ- and δ-lactam units that are present in three of the four top 200 small-molecule pharmaceuticals in terms of retail sales for the year 2022.^3^ The conformationally restricted lactam scaffold has been employed in peptidomimetic chemistry to improve potency, selectivity, and metabolic stability of peptide-based drugs.^4^

**Scheme 1:**
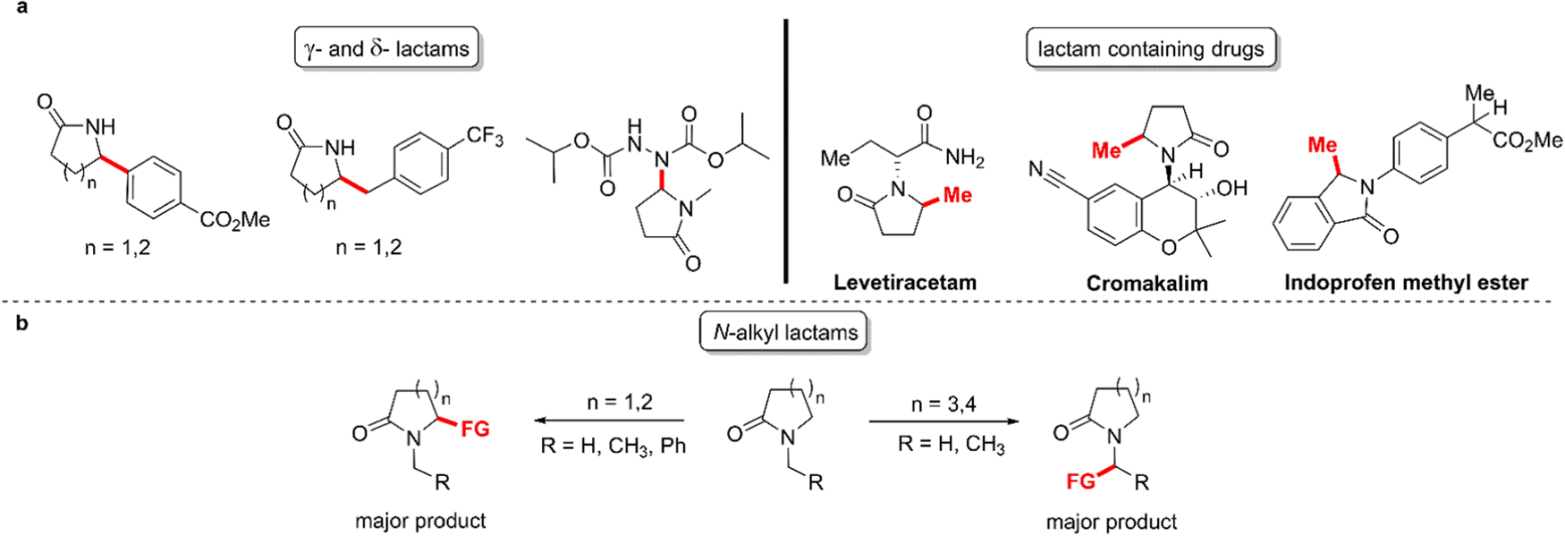
(a) Representative Examples of HAT-Based C(sp^3^)–H Bond Functionalization of Lactams and Lactam-Containing Drugs. (b) Competitive Endocyclic vs Exocyclic α-C–H Bond Functionalization in Tertiary Lactams

Synthetic approaches can offer the opportunity to access novel or modified derivatives of lead structures of pharmaceutical interest to be studied for improved potency or new activities. In the latter case, late-stage C(sp^3^)–H bond functionalization allows structural diversification by direct introduction of functional groups at specific sites without resorting to de novo synthesis, providing straightforward access to diversely functionalized derivatives with improved atom and step economy.^5^ Among the available strategies, nondirected hydrogen atom transfer (HAT) has emerged as a powerful tool for the functionalization of nitrogen-containing heterocycles.^6^ In the specific case of lactam substrates, HAT preferentially occurs from electron-rich C–H bonds α to nitrogen which are hyperconjugatively activated toward electrophilic reagents (Scheme 1, panel a).^7,8^

With *N*-alkyl lactams, HAT can, in principle, occur from both the endocyclic and exocyclic α-C–H bonds. 1-Alkyl-2-pyrrolidones have been mostly employed as substrate probes for site-selectivity studies. Within this substrate group, the 1-methyl, 1-ethyl, and 1-benzyl derivatives were shown to undergo predominant or exclusive functionalization at the endocyclic over exocyclic α-C–H bonds (Scheme 1, panel b, left).^9^ Similar trends were observed in the corresponding reactions of 1-alkyl-2-piperidone derivatives,^10^ whereas site selectivity was observed to shift toward the exocyclic α-C–H bonds with the 7- and 8-membered 1-alkylazepan-2-one and 1-alkylazocan-2-one derivatives (Scheme 1, panel b, right).^10b,10c,11^ In all of these studies, no explanation was provided to account for these selectivity patterns.

In this context, a recent kinetic study carried out by some of us on the reactions of 5- to 8-membered secondary and tertiary *N*-methyl lactams with the cumyloxyl radical (PhC(CH_3_)_2_O^•^, CumO^•^), a prototypical electrophilic HAT reagent, provided information on the effect of ring size on reactivity.^12^ Within each series, comparable reactivities were measured for the 5- and 6-membered ring derivatives. A decrease in reactivity with increasing ring size was then observed, approaching a factor ∼4 with the 8-membered azocan-2-one derivatives (see Table 1). This behavior was rationalized on the basis of the degree of hyperconjugative activation of the endocyclic α-C–H bonds imparted by the nitrogen center, which was proposed to decrease with increasing ring size and flexibility.

**Table 1:**
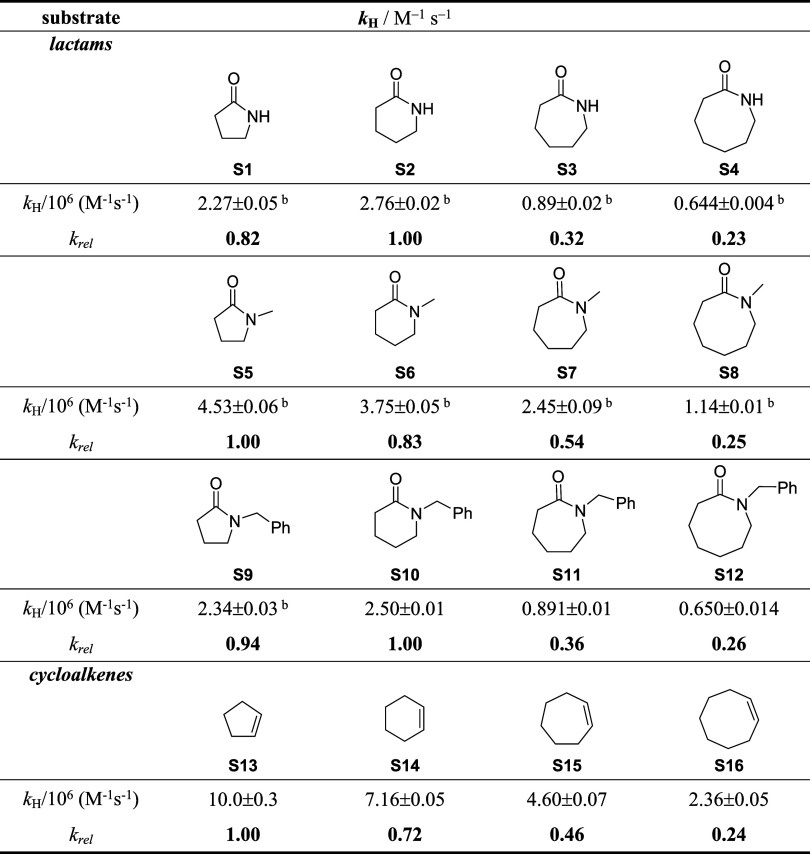
Second-Order Rate Constants (*k*_H_) for Reaction of the Cumyloxyl Radical (CumO^•^) with Lactams (**S1**–**S12**) and Cycloalkenes (**S13**–**S16**)^a^

a Measured in argon-saturated acetonitrile solution at *T* = 25 °C employing 355 nm LFP; [dicumyl peroxide] = 1.0 M.

b The results for substrates **S1**–**S9** are taken from ref (12).

In keeping with the importance of lactam structural motifs in natural products and pharmaceuticals, and with the paucity of 7- and 8-membered nitrogen heterocyclic units in medicinal chemistry libraries,^1d^,^13^ a point of particular interest that arises from these observations is the possibility of increasing ring size as a strategy to retard oxidative degradation pathways in lactam-containing drugs. The lower reactivity toward HAT reagents displayed by these medium-sized lactam cores may also offer the opportunity to promote late-stage diversification via otherwise unfavorable functionalization pathways at exocyclic α-C–H bonds.

To develop a deeper understanding of the reactivities and site selectivities observed in HAT from the C–H bonds of these structures, and to open the way to their possible synthetic applications, we conducted a detailed study on the reactions of *tert*-alkoxyl radicals with 5- to 8-membered secondary and tertiary *N*-methyl and *N*-benzyl lactams (substrates **S1**–**S12** in Table 1). The kinetic study on the reactions of CumO^•^, previously carried out for the former two substrate series,^12^ has been extended to the corresponding tertiary *N*-benzyl lactams, and these results have been accompanied by product studies on the reactions of all substrates with the *tert*-butoxyl radical ((CH_3_)_3_CO^•^, *t*BuO^•^). For comparison, a parallel study of the reactions of these two *tert*-alkoxyl radicals with 5- to 8-membered cycloalkenes (substrates **S13**–**S16** in Table 1) has been carried out. Complementary calculations of the bond dissociation enthalpies (BDEs) of the substrate C–H bonds involved in these reactions were also carried out.

## Results

### Kinetic Studies

CumO^•^ was generated following 355 nm laser flash photolysis (LFP) of argon-saturated acetonitrile solutions (*T* = 25 °C) containing 1.0 M dicumyl peroxide. In this solvent, CumO^•^ is characterized by a broad absorption band in the visible region of the spectrum centered at 485 nm. In the absence of added substrate, CumO^•^ decays mainly by C–CH_3_ β-scission.^14^ Addition of the lactam or cycloalkene substrate results in an increase in the CumO^•^ decay rate, and the observed rate constants (*k*_obs_) were determined following the decay of the visible absorption band as a function of the substrate concentration. When the *k*_obs_ values were plotted against substrate concentration, excellent linear relationships were obtained and the second-order rate constants for HAT to CumO^•^ (*k*_H_) were derived from the slope of these plots (see the Supporting Information for full details).

The *k*_H_ values measured in acetonitrile for the reaction of CumO^•^ with *N*-benzyl lactams (**S10**-**S12**) and cycloalkenes (**S13**-**S16**) are collected in Table 1. Also included in this table are the *k*_H_ values measured previously under analogous experimental conditions for reaction of CumO^•^ with secondary lactams (**S1**-**S4**), *N*-methyl lactams (**S5**-**S8**), and *N*-benzyl-pyrrolidin-2-one (**S9**).^12^

### Product Studies

**Scheme 2:**
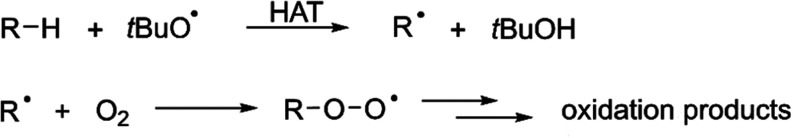
HAT-Based C(sp^3^)–H Bond Oxygenation Promoted by *t*BuO^•^

*t*BuO^•^ has been preferred to CumO^•^ as the HAT reagent for product studies.^15^ These two radicals have been shown to display similar HAT reactivities in their reactions with different substrates, but the significantly lower rate constant for C–CH_3_ β-scission measured for *t*BuO^•^ compared to CumO^•^ (in acetonitrile *k*_β_ = 6.4 × 10^4^ and 7.4 × 10^5^ s^–1^,^14^ respectively) makes competition with bimolecular HAT more favorable for the former radical. *t*BuO^•^ was generated by 310 nm steady-state photolysis of oxygen-saturated acetonitrile solutions containing di-*tert*-butyl peroxide (DTBP, 0.3–0.5 M), in the presence of the lactam or cycloalkene substrate (**S1**–**S16**, 0.1–0.2 M). Under these conditions, HAT from the substrate C(sp^3^)–H bonds to *t*BuO^•^ occurs, and the carbon radicals thus formed are rapidly trapped by oxygen, to give intermediate peroxyl radicals that evolve into the oxidation products (Scheme 2). No reaction product was observed in the absence of DTBP or light.

### Lactams

**Scheme 3:**
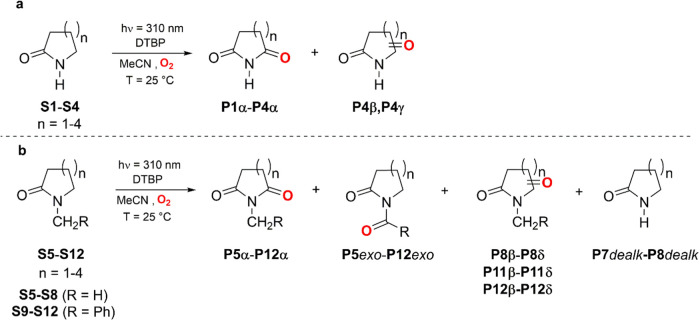
Structure of the Products Observed in the Oxygenation of (a) Secondary and (b) Tertiary Lactams Promoted by *t*BuO^•^

The reactions of *t*BuO^•^ with lactams **S1**–**S12** were carried out in acetonitrile at *T* = 25 °C, for irradiation times ranging between 1 and 18 h. Starting from the reactions of the secondary lactams, exclusive formation of the imide products (**P1α**–**P3α**) deriving from oxygenation at the carbon atom α to nitrogen was observed in the reactions of **S1–S3** (Scheme 3a). With **S4**, the formation of imide **P4α** was accompanied by smaller amounts of ketolactam products **P4β** and **P4γ** deriving from oxygenation at other ring positions. Full details of the results obtained in these experiments are reported in the Supporting Information (SI) (Table S1a,b).

With the tertiary *N*-methyl and *N*-benzyl lactams (**S5**–**S8** and **S9**–**S12**, respectively), competitive oxidation of the endocyclic and exocyclic ring positions α to the nitrogen atom was observed in all cases, leading to imide (**P5α**–**P12α**) and acyl lactam (**P5***exo*–**P12***exo*) products, respectively (Scheme 3b). In the reactions of **S8**, **S11**, and **S12**, the formation of the ketolactam products (**P8β-P8δ**, **P11β-P11δ**, and **P12β-P12δ**, respectively) deriving from oxygenation at β, γ, and δ ring positions was also observed. With **S7** and **S8**, small amounts of the corresponding demethylated secondary lactam (**P7***dealk* = **S3** and **P8***dealk* = **S4**) were also detected among the reaction products.^15^ Full details of the product distributions observed in the oxidation of **S5–S12** promoted by *t*BuO^•^ are displayed in the SI (Tables S2–S9).

**Figure 1:**
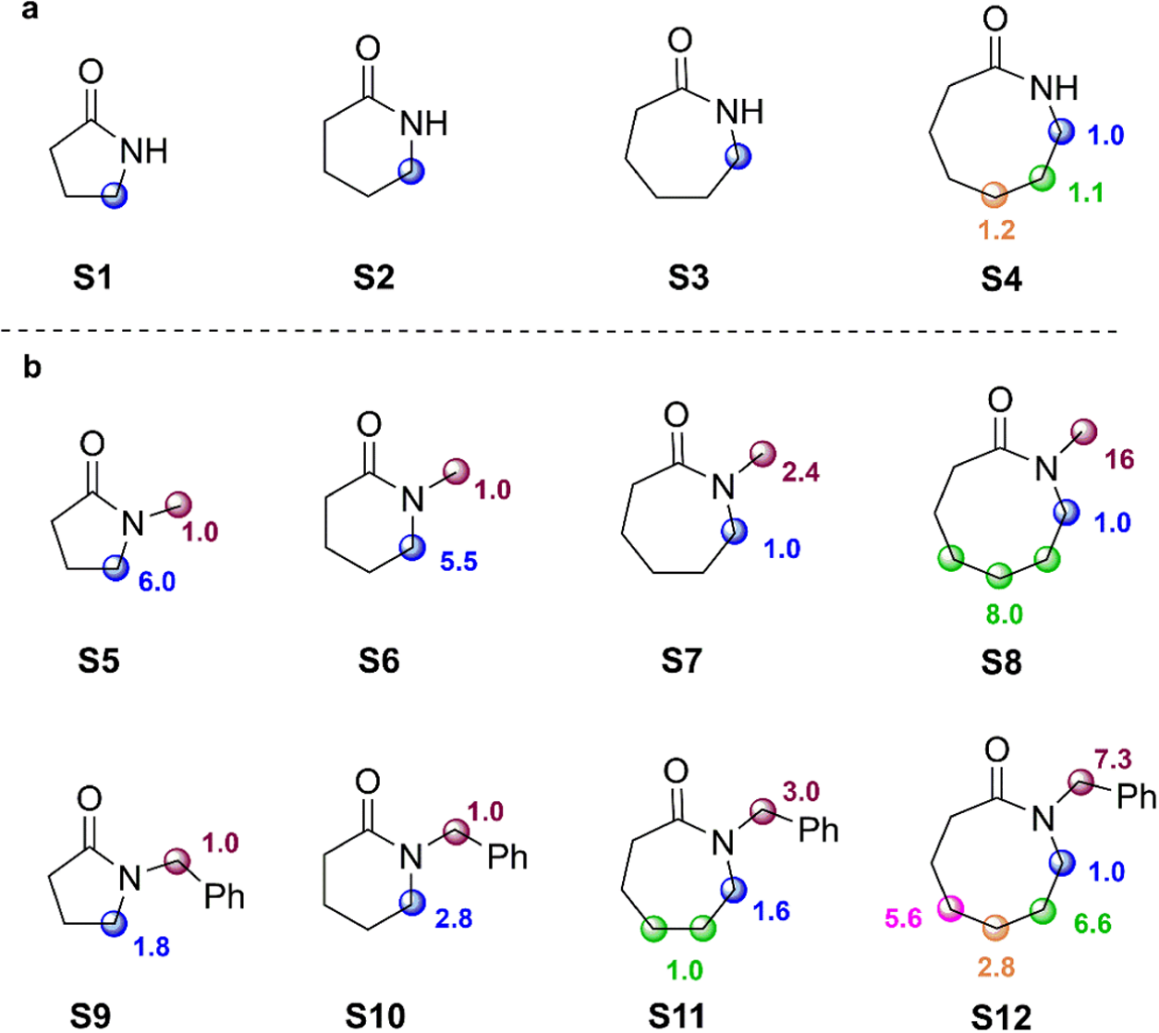
Product distributions obtained in the oxygenation of (a) secondary (**S1**–**S4**) and (b) tertiary lactams (**S5**–**S12**) promoted by *t*BuO^•^.

The relative product distributions, obtained in the oxygenation of lactams **S1**–**S12** promoted by *t*BuO^•^, are displayed in Figure 1.^16^

### Cycloalkenes

**Scheme 4:**
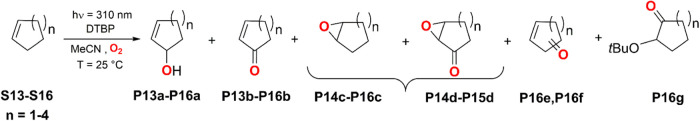
Structure of the Products Observed in the Oxygenation of Cycloalkenes **S13**–**S16** Promoted by *t*BuO^•^

With the cycloalkene substrates **S13**–**S16**, the reactions with DTBP were carried out in oxygen-saturated acetonitrile at *T* = 25 °C, for irradiation times between 5 and 120 min. The structures of the products observed in these reactions are displayed in Scheme 4. Full details of the results obtained in all of these experiments are reported in the SI (Tables S10–S13).

**Figure 2:**
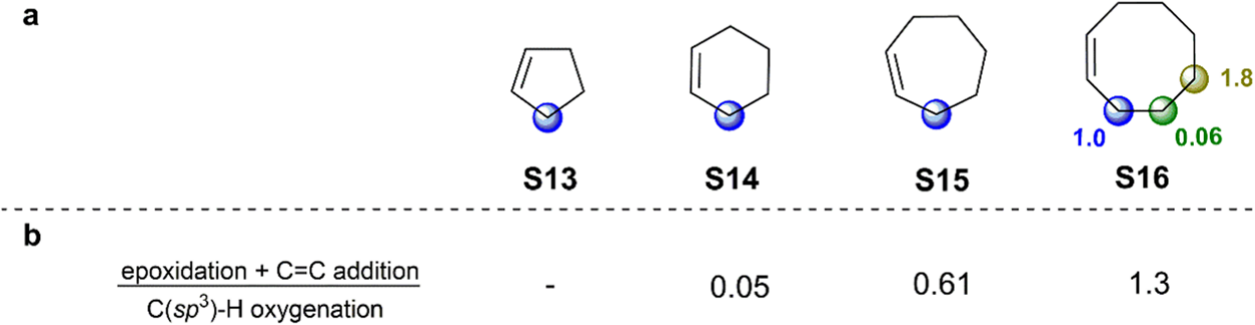
(a) Product distributions obtained in the oxygenation of cycloalkenes (**S13**–**S16**) promoted by *t*BuO^•^. (b) Relative importance of the products deriving from epoxidation and C=C bond addition over those deriving from C(sp^3^)–H bond hydroxylation and ketonization.

Reaction of *t*BuO^•^ with **S13**–**S16** led in all cases to the formation of the allylic alcohol (**P13a**–**P16a**) accompanied by the corresponding 2-cycloalkenone (**P13b**–**P16b**). With **S14**–**S16**, cycloalkene and cycloalkenone oxides (**P14c**–**P16c** and **P14d**–**P15d**, respectively) were also observed among the reaction products. In the reaction of **S16**, formation of 4-cyclooctenone (**P16e**), 3-cyclooctenone (**P16f**, in trace amounts), and 2-(*tert*-butoxy)-cyclooctan-1-one (**P16g**) was also observed. In analogy with the results obtained for the lactam substrates, the relative product distributions for C(sp^3^)–H bond hydroxylation and ketonization of cycloalkenes **S13**-**S16** promoted by *t*BuO^•^, are displayed in Figure 2a.

Figure 2b highlights the relative importance of the competitive reaction pathways, expressed in terms of the ratio between the sum of the products derived from epoxidation and C=C bond addition and those derived from C(sp^3^)–H bond hydroxylation and ketonization (see below).

### Computational Results

**Figure 3:**
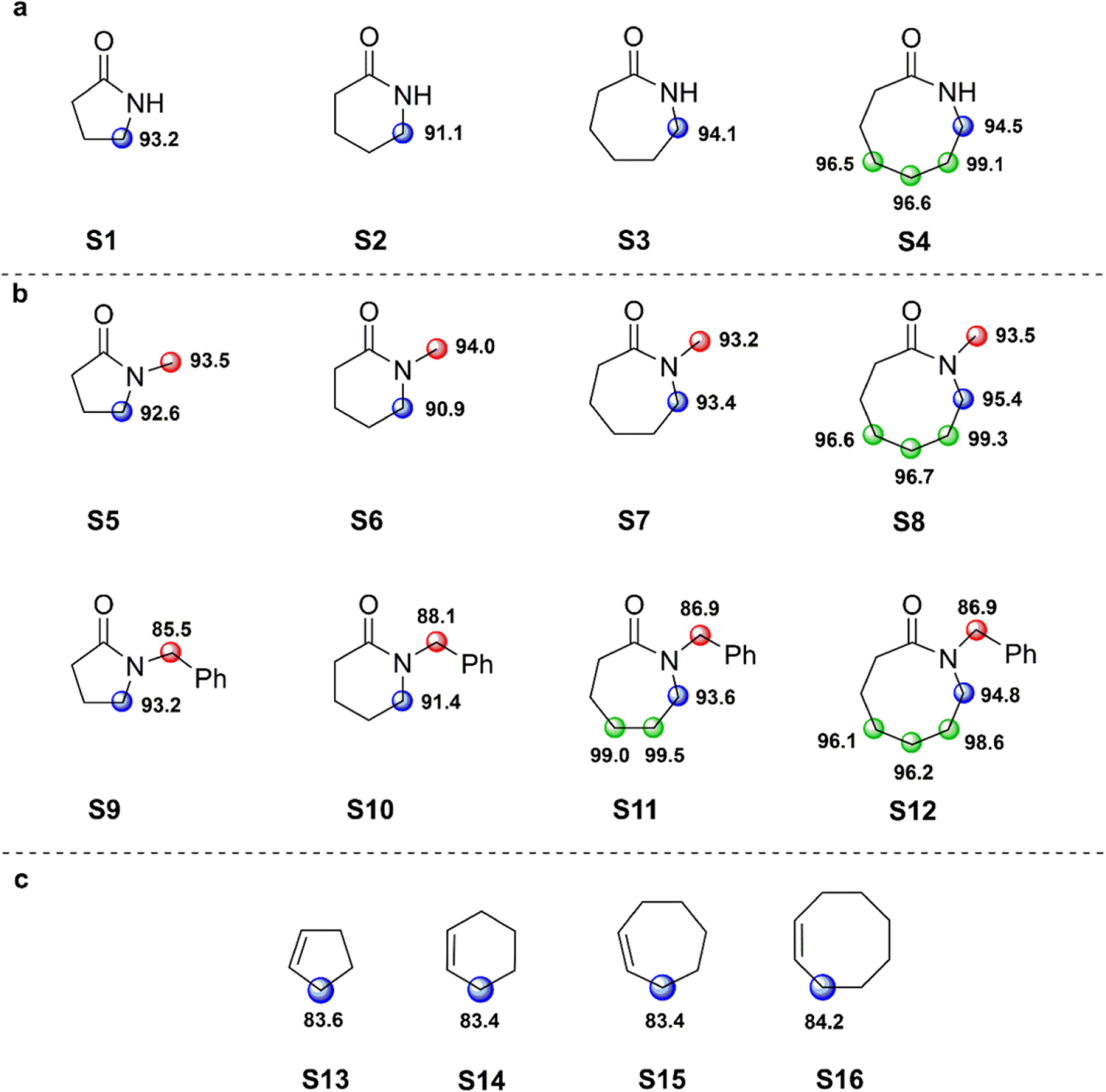
Calculated BDE values ((RO)CBS-QB3) for the relevant C–H bonds of (a) secondary lactams **S1–S4**; (b) tertiary lactams **S5–S12**; (c) cycloalkenes **S13–S16**. The given values are in kcal mol^–1^.

For the 7- and 8-membered ring lactam and alkene parent molecules, comprehensive conformer searches were performed using the ConfBuster software.^17^ All conformers generated by ConfBuster were used to create the radical structures by removing a hydrogen atom at each of the positions indicated in Figure 3. All conformers were subjected to geometry optimization using the ωB97XD^18^/pcseg-1^19^-ACP approach, a method that was specifically designed to predict accurate BDEs at low computational cost.^20^ The parent and radical structures with the lowest electronic energies from the ACP approach were retained for BDE calculations using the (RO)CBS-QB3 method^21^ and are displayed in Figure 3. Our previous work has demonstrated that (RO)CBS-QB3 produces gas-phase BDEs that are within ca. 1 kcal/mol of the true values.^22^ BDE calculations were performed using the Gaussian 16 program package.^23^ The ωB97XD/pcseg-1-ACP BDEs, which are presented in the SI (Figure S8), were found to have a mean absolute deviation of about 0.7 kcal/mol relative to the (RO)CBS-QB3 BDEs.

## Discussion

**Figure 4:**
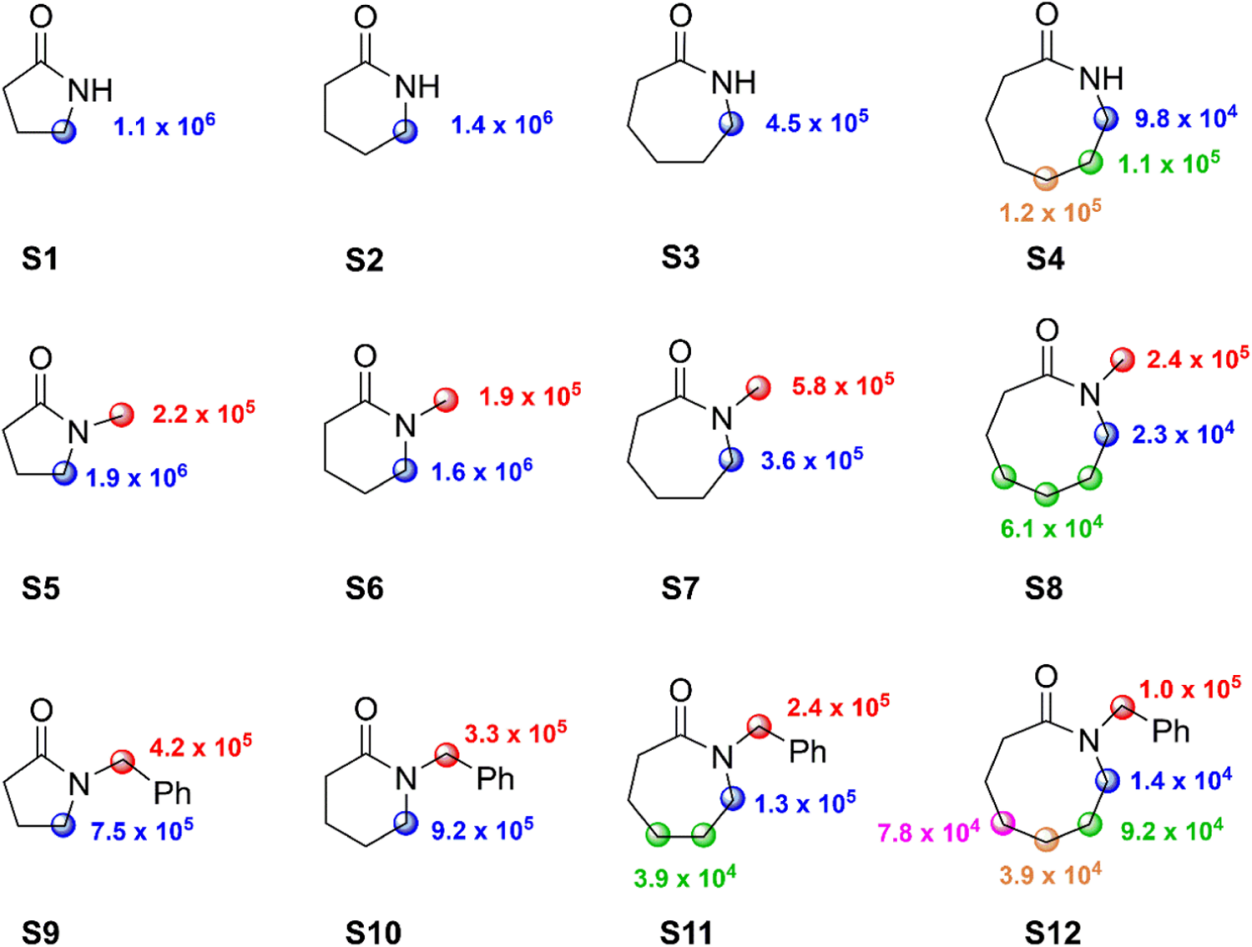
Normalized rate constants (*k*_H_(norm) in M^–1^ s^–1^) for HAT from the C–H bonds of substrates **S1**–**S12** to *tert*-alkoxyl radicals.

The measured *k*_H_ values for the reaction of CumO^•^ with *N*-benzyl lactams **S9**-**S12**, displayed in Table 1, show a reactivity trend that is very similar to those observed previously for the corresponding reactions of secondary (**S1**–**S4**) and tertiary *N*-methyl lactams (**S5**–**S8**).^10^ Within this series, similar values were measured for the 5- and 6-membered derivatives **S9** and **S10**, with *k*_H_ values that decrease with increasing ring size, approaching a ∼4-fold decrease in reactivity with the 8-membered derivative **S12**. We derived the normalized rate constants (*k*_H_(norm)) for HAT from the different C–H bonds of **S1**–**S12** by combining the normalized site selectivities for C–H bond oxygenation observed in the reactions of *t*BuO^•^ (Figure 1) with the corresponding *k*_H_ values measured for the reaction with CumO^•^ (Table 1). The *k*_H_(norm) values thus obtained are collected in Figure 4.^24,25^

This combined analysis shows that in the secondary lactam series, the *k*_H_(norm) value for HAT from the C–H bonds α to nitrogen decreases by a factor ∼3 going from the 5- and 6-membered derivatives **S1** and **S2** to the 7-membered derivative **S3**, and by a factor of up to 15 going to the 8-membered derivative **S4**. This trend is also roughly reflected in the calculated α-C–H BDEs displayed in Figure 3 that, although differ by 2.1 kcal mol^–1^ between **S1** and **S2**, increase for both substrates going to **S3** and **S4**. Interestingly, with **S4**, the significantly larger decrease in α-C–H reactivity translates into competitive oxygenation at different ring sites with imide **P4α** deriving from α-C–H bond oxygenation, and ketolactams **P4β** and **P4γ** formed following oxygenation at the β- and γ-C–H bonds that are formed in comparable amounts. The **P4α**/(**P4β** + **P4γ**) product ratio was determined as 1:2.3 (Figure 1).

Similar trends were observed in the corresponding reactions of the tertiary *N*-methyl and *N*-benzyl lactams **S5**–**S8** and **S9**–**S12**, for which the *k*_H_(norm) values for HAT from the endocyclic α-C–H bonds decreased by factors up to 5 and ∼80 for the former series and up to 7 and ∼60 for the latter one, going from the 5- and 6-membered derivatives to the 7- and 8-membered ones. These findings are again roughly aligned with the calculated BDEs.

Lactams **S5**–**S12** also bear exocyclic α-C–H bonds that can compete with the other sites for HAT to the *tert*-alkoxyl radicals. Along this line, the product distributions and associated *k*_H_(norm) values displayed in Figures 1 and 4, respectively, clearly show that site selectivity progressively shifts from the endocyclic to the exocyclic α-C–H bonds, going from the 5- and 6-membered derivatives to the 7- and 8-membered ones, a behavior that aligns with the results of previous studies on HAT-based functionalization of tertiary lactams.^9−11^ Interestingly, while the BDEs for the endocyclic α-C–H bonds increase with increasing ring size on going from **S6** to **S8** and from **S10** to **S12**, those for the exocyclic α-C–H bonds do not change significantly along the two series. Accordingly, with the exception of **S7**, very similar *k*_H_(norm) values for HAT from the exocyclic α-C–H bonds of the *N*-methyl lactams were derived, and an ∼4-fold decrease in *k*_H_(norm) was observed along the *N*-benzyl lactam series. Taken together, these results are indicative of a strong deactivation toward HAT of the endocyclic α-C–H bonds of both substrate groups and a weaker one for the benzylic α-C–H bonds, associated with an increase in ring size that translates into competitive oxygenation at different endocyclic sites for the *N*-methyl and *N*-benzyl derivatives **S8**, **S11**, and **S12**, with the contribution of these pathways that becomes dominant with the latter substrate.

The relatively low benzylic α-C–H bond reactivity of substrates **S9**–**S12** deserves special consideration. Similar reactivity trends have been recently observed in the reactions of CumO^•^ with *N*-alkyl and *N*,*N*-dialkylalkanamides, where comparable *k*_H_ values have been measured for HAT from the α-C–H bonds of *N*-methyl- and *N*-benzylacetamide (*k*_H_ = 2.9 × 10^5^ and 5.2 × 10^5^ M^–1^ s^–1^, respectively), and a 7-fold decrease in *k*_H_ has been measured going from *N*,*N*-dimethyl- to *N*,*N*-dibenzylacetamide (*k*_H_ = 1.5 × 10^6^ and 2.1 × 10^5^ M^–1^ s^–1^, respectively), despite the significantly lower BDEs associated with the benzylic α-C–H bonds.^15^ This behavior has been rationalized in terms of the operation of steric and stereoelectronic effects, where structural features preclude the attainment of a conformation in which the benzylic α-C–H bond is perpendicular to the planes of both the amide and phenyl moieties. A lack of benzylic activation has been also observed in kinetic studies on the reactions of CumO^•^ with different C(sp^3^)–H bond donors, where the measured *k*_H_ values for HAT from benzylic and allylic C–H bonds were significantly lower than what would have been expected on the basis of their BDEs.^22^ The behavior was explained in terms of Bernasconi’s principle of nonperfect synchronization (PNS), with the relative importance of benzylic or allylic resonance stabilization that develops late along the reaction coordinate and contributes to a limited extent to the stabilization of the HAT transition state.^26,27^ In keeping with these previous studies, it is reasonable to assume that the lack of benzylic activation observed in the reactions of *N*-benzyl lactams **S9**–**S12** can be rationalized accordingly in terms of the operation of steric and stereoelectronic effects, as well as of the above-mentioned kinetic penalty associated with delayed resonance stabilization.

The similar trends observed along the secondary and tertiary lactam series indicate that endocyclic α-C–H bond deactivation associated with a ring-size increase is an intrinsic feature of these substrates. These observations support the hypothesis that the greater flexibility of medium-sized rings decreases the degree of hyperconjugative α-C–H bond activation imparted by the nitrogen center, leading to bond strengthening, which, in turn, accounts for the observed selectivity patterns. This hypothesis is nicely supported by the results of Natural Bond Orbital (NBO) calculations, carried out for the secondary and tertiary *N*-methyl and *N*-benzyl lactam series (and for the cycloalkene series), that are displayed in the SI (Figure S9). Comparison between the *k*_H_(norm) values for HAT from the endocyclic α-C–H bonds of secondary and tertiary lactams having the same ring size provides information on the role of the *N*-substituent in reactivity. Within the 5- and 6-membered derivatives, a slight increase in *k*_H_(norm) has been observed going from the secondary to the *N*-methyl derivatives, a behavior that may reflect an increase in α-C–H bond electron density determined by alkyl substitution.^12^ This activating effect is lost for the corresponding 7- and 8-membered derivatives, where a decrease in *k*_H_(norm) was observed. Different behavior was observed for the *N*-benzyl derivatives, where *k*_H_(norm) for HAT from the endocyclic α-C–H bonds is always lower than those derived for the corresponding secondary and tertiary *N*-methyl lactams, indicating that other effects associated with the replacement of a methyl hydrogen atom by phenyl override this activating electronic effect. In addition to the reduced extent of hyperconjugation discussed above, steric and torsional effects associated with the presence of the phenyl group and with the planarization of the incipient carbon radical in the transition state for HAT from the endocyclic α-C–H bond may account for the observed behavior.^28^

In the context of the foregoing discussion, Table 2 compares the *k*_H_(norm) values for HAT from the endocyclic α-C–H bonds of secondary and tertiary lactams **S1**-**S12** with those determined previously for HAT from the C–H bonds of the corresponding cycloalkanes to CumO^•^.^22^

**Table 2:**
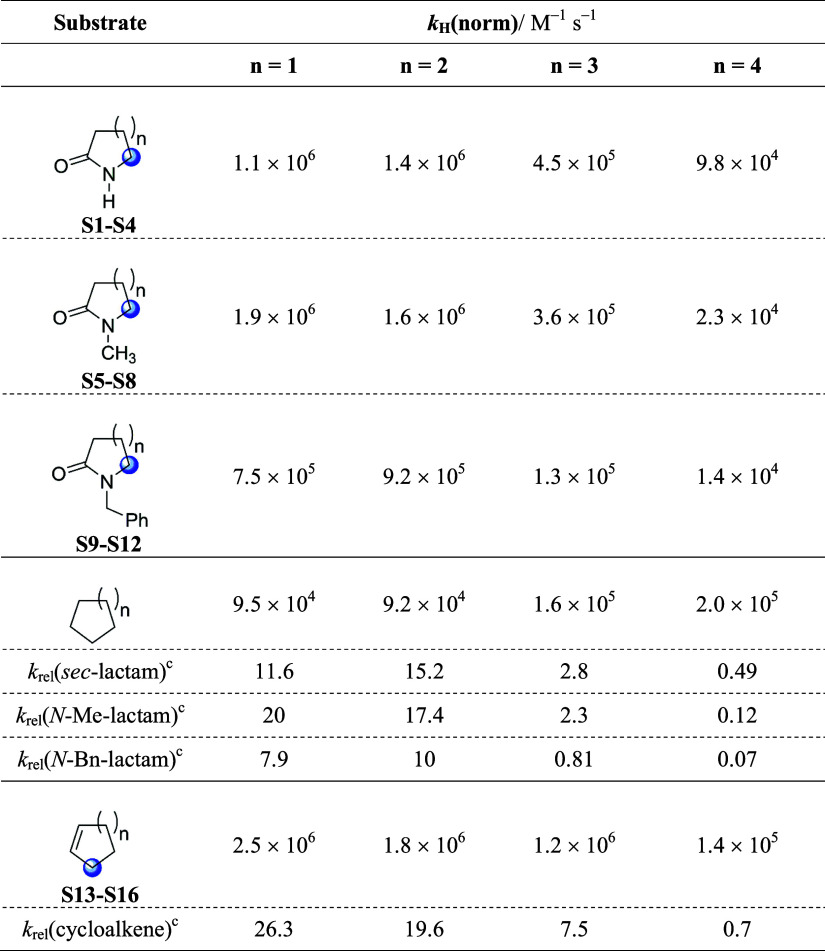
Normalized Rate Constants (*k*_H_(norm)) for HAT from the Endocyclic α-C–H Bonds of Secondary and Tertiary Lactams **S1**–**S12**,^a^ from the C–H Bonds of Cycloalkanes,^b^ and from the α-C–H Bonds of Cycloalkenes **S13**–**S16**^a^ to *tert*-Alkoxyl Radicals

a This work (see Figure 4).

b Ref (22).

c *k*_rel_ is the ratio between *k*_H_(norm) for a given lactam or cycloalkene substrate and *k*_H_(norm) for the corresponding cycloalkane.

Most interestingly, while the α-C–H bonds of lactams **S1** and **S2** are strongly activated compared to the C–H bonds of cyclopentane and cyclohexane (*k*_rel_ = 11.6 and 15.2, respectively), this activation decreases with increasing ring size and fades away with **S4** for which the α-C–H bonds shows a *k*_H_(norm) value that is smaller by a factor ∼2 compared to the C–H bonds of cyclooctane (*k*_rel_ = 0.49). As shown in Figure 4, such deactivation also extends to the β- and γ-C–H bonds, a behavior that can be accounted for on the basis of the deactivating electronic effect exerted by the remote carbonyl group. The same trends are also observed when extending the comparison to tertiary lactams, where, however, and most importantly, the presence of an *N*-methyl or *N*-benzyl substituent determines a significantly larger decrease in *k*_rel_ when comparing 8-membered lactams **S8** and **S12** with cyclooctane (*k*_rel_ = 0.12 and 0.07, respectively), indicating that α-C–H bond deactivation is amplified by the introduction of *N*-substituents.

It is also very interesting to compare the reactivity and selectivity patterns observed in the reactions of *tert*-alkoxyl radicals with lactams **S1**–**S12**, with those observed for the corresponding reactions of cycloalkenes **S13**–**S16**. The *k*_H_ values for reaction with CumO^•^, displayed in Table 1, show again a reactivity trend that is very similar to those observed for the three lactam series, with *k*_H_ that decreases with increasing ring size, approaching an ∼4-fold decrease in reactivity going from cyclopentene (**S13**) to cyclooctene (**S16**). These values include, however, the contribution of all bimolecular reaction pathways that are responsible for CumO^•^ decay. The contribution of the HAT pathway to the measured *k*_H_ value can be derived from the product distributions obtained in the oxygenation of **S13**–**S16** promoted by *t*BuO^•^ displayed in Scheme 4 and SI. With **S13**, exclusive formation of 2-cyclopentenol (**P13a**) and 2-cyclopentenone (**P13b**) was observed (see the SI, Table S10). Formation of these products can be explained in terms of a reaction initiated by HAT from the allylic C–H bonds to *t*BuO^•^ to give a carbon radical that is rapidly trapped by oxygen.^29^ The intermediate peroxyl radical thus formed evolves, leading to the formation of **P13a** and **P13b**.^15,30^ Overoxidation of **P13a** may also contribute to the formation of **P13b**. With **S14**–**S16**, formation of the 2-cycloalkenol (**P14a**–**P16a**) and 2-cycloalkenone (**P14b**–**P16b**) products was also observed, accompanied however by the corresponding cycloalkene and cycloalkenone oxides (**P14c**–**P16c** and **P14d**–**P15d**, respectively) and, in the specific case of **S16**, by 4-cyclooctenone (**P16e**), 3-cyclooctenone (**P16f**), and 2-(*tert*-butoxy)-cyclooctanone (**P16g**) (see SI, Tables S11–S13).

Formation of the epoxidation products can be explained on the basis of a previously proposed mechanism through addition of a peroxyl radical to the C=C double bond, followed by intramolecular homolytic substitution on the peroxide bond with release of an alkoxyl radical (see SI, Scheme S3), indicating that formation of these products does not contribute to the decay of CumO^•^.^31,32^ Experiments carried out subjecting 2-cyclohexenone **P14b** and cyclohexene oxide **P14c** to the reaction conditions (see SI, Scheme S5) showed that formation of 2-cyclohexenone oxide **P14d** was only observed in the former case, providing strong support to the hypothesis that cycloalkenone oxides **P14d**–**P15d** derive from the epoxidation of the first formed 2-cycloalkenones **P14b**–**P15b**, and not from ketonization of cycloalkene oxides **P14c**–**P15c**. Formation of **P16g** can be instead rationalized in terms of the addition of *t*BuO^•^ (or CumO^•^, see SI, Table S13) to the C=C bond of **S16**, followed by oxygen trapping (see SI, Scheme S6), indicating that the formation of this product contributes to the decay of CumO^•^.

Further support to the contribution of the competitive epoxidation and *tert*-butoxyl addition pathways was provided by the results of oxygenation experiments carried out on norbornene (**S17**) and styrene (**S18**), substrates that lack C–H bonds that are activated toward HAT to *tert*-alkoxyl radicals and are therefore customarily employed as probes for the study of radical C=C bond addition reactions.^33^ Full details of these experiments are described in the SI (Tables S14 and S15, respectively).

On the basis of these findings, the normalized distribution for hydroxylation and ketonization products deriving from HAT from the C–H bonds of cycloalkenes **S13**-**S16** to *t*BuO^•^ is displayed in Figure 2 together with the ratio of products deriving from HAT and from competitive pathways (epoxidation and C=C addition). The lack of epoxidation and *tert*-butoxyl addition products in the reaction of **S13**, accompanied by the observation that the relative amount of these products increases with increasing ring size, clearly indicates that within the cycloalkene series, the rate constant for HAT from the most activated allylic C–H bonds decreases with increasing ring size. This observation is also well supported by the formation, in the reaction of **S16**, of products **P16e** and **P16f**, derived from competitive HAT-based oxygenation at more remote β- and γ-C–H bonds. By combining the normalized product distributions displayed in Figure 2, with the corresponding *k*_H_ values for reaction with CumO^•^ collected in Table 1, and taking into account that the *k*_H_ value measured for **S16** also includes the contribution of *tert*-alkoxyl radical addition to the C=C bond, the normalized rate constants (*k*_H_(norm)) for HAT from the α-C–H bonds of these substrates to *tert*-alkoxyl radicals were derived. These values are also included in Table 2 and show that *k*_H_(norm) progressively decreases with increasing ring size going from 2.5 × 10^6^ M^–1^ s^–1^ for **S13** to 1.4 × 10^5^ M^–1^ s^–1^ for **S16**. In keeping with the foregoing discussion on the comparison between the *k*_H_(norm) values for HAT from the α-C–H bonds of lactams and cycloalkanes, the derived rate constants show that the allylic α-C–H bonds of cyclopentene **S13** and cyclohexene **S14** are strongly activated compared to the C–H bonds of cyclopentane and cyclohexane (*k*_rel_ = 26.3 and 19.6, respectively) and that this activation is no longer observed with **S16** for which the α-C–H bond shows a *k*_H_(norm) value that is slightly lower than the one derived for cyclooctane (*k*_rel_ = 0.7), despite a difference in BDE of about 10 kcal mol^–1^.^22^

The similar reactivity trends observed along the lactam and cycloalkene series, quantified by the *k*_H_(norm) values displayed in Table 2, together with the significantly smaller α-C–H BDE difference within the cycloalkene series compared to the lactam ones (ΔBDE = 0.8 and 3.4–4.5 kcal mol^–1^, respectively) indicate that the origin of these effects is mostly kinetic, with the stability of the intermediate carbon radical that plays a minor role. Along this line, the observation that the *k*_H_(norm) values for HAT from the cycloalkene α-C–H bonds are higher, within a factor 10, than those derived for HAT from the endocyclic α-C–H bonds of the corresponding lactams, despite differences in BDE that exceed in all cases 7 kcal mol^–1^ (Figure 3), represents an additional manifestation of the above-mentioned Bernasconi’s PNS.

In conclusion, these results clearly indicate that in HAT reactions to *tert*-alkoxyl radicals, similar factors, associated with an increase in ring size, account for the strong α-C–H bond deactivation in lactams and cycloalkenes. Decreases in reactivity that approach factors of 83 and 18 have been measured for HAT from the endocyclic α-C–H bonds of tertiary *N*-methyl lactams and from the allylic C–H bonds of cycloalkenes, respectively, going from the 5- and 6-membered derivatives to the 8-membered ones. These differences in reactivity translate, in the tertiary *N*-methyl and *N*-benzyl lactams, in a change in site selectivity, with the relative importance of HAT from the exocyclic α-C–H bonds that increase with increasing ring size. The results support the hypothesis that the greater flexibility of the medium-sized rings determines a decrease in the extent of hyperconjugative overlap with the amide or C=C π-system, increasing the kinetic barrier associated with the HAT step. We envisage that these findings may offer the opportunity to finely tune the properties of lactam-containing drugs through changes in ring size and *N*-substitution, providing access to new derivatives of pharmaceutical leads to be screened in medicinal chemistry campaigns.

## Data Availability

The data underlying this study are available in the published article and its Supporting Information.

## Supporting Information Available

Experimental section and additional experimental data, including NMR spectra and computational details (PDF)
BDEs for lactams and cycloalkenes (XYZ) (ZIP)

## References

[ref1] aHeravi M. M.; Zadsirjan V. Prescribed drugs containing nitrogen heterocycles: an overview. RSC Adv. 2020, 10, 44247–44311. 10.1039/D0RA09198G. 35557843 PMC9092475

[ref2] aSaldívar-González F. I.; Lenci E.; Trabocchi A.; Medina-Franco J. L. Exploring the chemical space and the bioactivity profile of lactams: a chemoinformatic study. RSC Adv. 2019, 9, 27105–27116. 10.1039/C9RA04841C. 35528563 PMC9070607

[ref3] a2025 https://bpb-us-e2.wpmucdn.com/sites.arizona.edu/dist/9/130/files/2023/11/NjardarsonGroup2022SmallMoleculeTopPosterV3.pdf. (accessed March 3, 2025).

[ref4] Ung P.; Winkler D. A. Tripeptide motifs in biology: targets for peptidomimetic design. J. Med. Chem. 2011, 54, 1111–1125. 10.1021/jm1012984. 21275407

[ref5] aBellotti P.; Huang H.-M.; Faber T.; Glorius F. Photocatalytic late-stage C–H functionalization. Chem. Rev. 2023, 123, 4237–4352. 10.1021/acs.chemrev.2c00478. 36692361

[ref6] aCapaldo L.; Ravelli D.; Fagnoni M. Direct photocatalyzed hydrogen atom transfer (HAT) for aliphatic C–H bonds elaboration. Chem. Rev. 2022, 122, 1875–1924. 10.1021/acs.chemrev.1c00263. 34355884 PMC8796199

[ref7] aShu X.; Zhong D.; Huang Q.; Huan L.; Huo H. Site- and enantioselective cross-coupling of saturated N-heterocycles with carboxylic acids by cooperative Ni/photoredox catalysis. Nat. Commun. 2023, 14, 125 10.1038/s41467-023-35800-0. 36624097 PMC9829739

[ref8] Garwood J. J. A.; Chen A. D.; Nagib D. A. Radical Polarity. J. Am. Chem. Soc. 2024, 146, 28034–28059. 10.1021/jacs.4c06774. PMC12129049 39363280

[ref9] aWan T.; Wen Z.; Laudadio G.; Capaldo L.; Lammers R.; Rincón J. A.; García-Losada P.; Mateos C.; Frederick M. O.; Broersma R.; Noël T. Accelerated and Scalable C(sp^3^)–H Amination via Decatungstate Photocatalysis Using a Flow Photoreactor Equipped with High-Intensity LEDs. ACS Cent. Sci. 2022, 8, 51–56. 10.1021/acscentsci.1c01109. 35106372 PMC8796300

[ref10] aConiglio A.; Galli C.; Gentili P.; Vadalà R. Oxidation of amides by laccase-generated aminoxyl radicals. J. Mol. Catal. B: Enzym. 2008, 50, 40–49. 10.1016/j.molcatb.2007.09.022.

[ref11] aZhou J.; Ren Q.; Xu N.; Wang C.; Song S.; Chen Z.; Li J. K_2_S_2_O_8_-catalyzed highly regioselective amidoalkylation of diverse N-heteroaromatics in water under visible light irradiation. Green Chem. 2021, 23, 5753–5758. 10.1039/D1GC02107A.

[ref12] Galeotti M.; Trasatti C.; Sisti S.; Salamone M.; Bietti M. Factors Governing Reactivity and Selectivity in Hydrogen Atom Transfer from C(sp^3^)–H Bonds of Nitrogen-Containing Heterocycles to the Cumyloxyl Radical. J. Org. Chem. 2022, 87, 7456–7463. 10.1021/acs.joc.2c00955. 35609878 PMC9171822

[ref13] aMykura R.; Sánchez-Bento R.; Matador E.; Duong V. K.; Varela A.; Angelini L.; Carbajo R. J.; Llaveria J.; Ruffoni A.; Leonori D. Synthesis of polysubstituted azepanes by dearomative ring expansion of nitroarenes. Nat. Chem. 2024, 16, 771–779. 10.1038/s41557-023-01429-1. 38273027

[ref14] Salamone M.; Bietti M. Reaction pathways of alkoxyl radicals. The role of solvent effects on C–C bond fragmentation and hydrogen atom transfer reactions. Synlett 2014, 25, 1803–1816. 10.1055/s-0033-1341280.

[ref15] Sisti S.; Ioele F.; Scarchilli F.; Galeotti M.; DiLabio G. A.; Salamone M.; Bietti M. Activation and Deactivation of Benzylic C–H Bonds Guided by Stereoelectronic Effects in Hydrogen Atom Transfer from Amides and Amines to Alkoxyl Radicals. Eur. J. Org. Chem. 2023, 26, e202300419 10.1002/ejoc.202300419.

[ref16] Ketolactam products P4β, P4γ, and P12β-P12δ, have been isolated allowing quantification of the different isomers. Ketolactam products P8β-P8δ and P11β-P11δ have not been isolated and the values displayed in Figure 1 are expressed as the sum of the three isomers. For details see the SI.

[ref17] Barbeau X.; Vincent A. T.; Lagüe P. ConfBuster: Open-source tools for macrocycle conformational search and analysis J. Open Res. Software 2018, 6 (1), 10.5334/jors.189.

[ref18] Chai J.-D.; Head-Gordon M. Long-range corrected hybrid density functionals with damped atom–atom dispersion corrections. Phys. Chem. Chem. Phys. 2008, 10, 6615–6620. 10.1039/b810189b. 18989472

[ref19] Jensen F. *J*. Unifying general and segmented contracted basis sets. Segmented polarization consistent basis sets. Chem. Theory Comput. 2014, 10, 1074–1085. 10.1021/ct401026a. 26580184

[ref20] Prasad V. K.; Otero-de-la-Roza A.; DiLabio G. A. Bridging the gap between high-level quantum chemical methods and deep learning models. Mach. Learn.: Sci. Technol. 2024, 5, 015035 10.1088/2632-2153/ad27e1.

[ref21] Wood G. P. F.; Radom L.; Petersson G. A.; Barnes E. C.; Frisch M. J.; Montgomery J. A. Jr. A restricted-open-shell complete-basis-set model chemistry. J. Chem. Phys. 2006, 125, 094106 10.1063/1.2335438. 16965071

[ref22] Salamone M.; Galeotti M.; Romero-Montalvo E.; van Santen J. A.; Groff B. D.; Mayer J. M.; DiLabio G. A.; Bietti M. Bimodal Evans–Polanyi Relationships in Hydrogen Atom Transfer from C(sp^3^)–H Bonds to the Cumyloxyl Radical. A Combined Time-Resolved Kinetic and Computational Study. J. Am. Chem. Soc. 2021, 143, 11759–11776. 10.1021/jacs.1c05566. 34309387 PMC8343544

[ref23] Frisch M. J.; Trucks G. W.; Schlegel H. B.; Scuseria G. E.; Robb M. A.; Cheeseman J. R.; Scalmani G.; Barone V.; Petersson G. A.; Nakatsuji H.; Li X.; Caricato M.; Marenich A. V.; Bloino J.; Janesko B. G.; Gomperts R.; Mennucci B.; Hratchian H. P.; Ortiz J. V.; Izmaylov A. F.; Sonnenberg J. L.; Williams-Young D.; Ding F.; Lipparini F.; Egidi F.; Goings J.; Peng B.; Petrone A.; Henderson T.; Ranasinghe D.; Zakrzewski V. G.; Gao J.; Rega N.; Zheng G.; Liang W.; Hada M.; Ehara M.; Toyota K.; Fukuda R.; Hasegawa J.; Ishida M.; Nakajima T.; Honda Y.; Kitao O.; Nakai H.; Vreven T.; Throssell K.; Montgomery J. A. Jr.; Peralta J. E.; Ogliaro F.; Bearpark M. J.; Heyd J. J.; Brothers E. N.; Kudin K. N.; Staroverov V. N.; Keith T. A.; Kobayashi R.; Normand J.; Raghavachari K.; Rendell A. P.; Burant J. C.; Iyengar S. S.; Tomasi J.; Cossi M.; Millam J. M.; Klene M.; Adamo C.; Cammi R.; Ochterski J. W.; Martin R. L.; Morokuma K.; Farkas O.; Foresman J. B.; Fox D. J. Gaussian 16, Revision C.01; Gaussian, Inc.: Wallingford, CT, 2016.

[ref24] A reviewer suggested that intermediate peroxyl radicals, generated after O_2_ trapping of the first formed carbon radicals, could also promote additional C–H oxidation events. The significantly higher HAT reactivity of alkoxyl compared to alkylperoxyl radicals (for toluene *k*_HAT_ = 2 × 10^5^ and 1 × 10^–2^ M^–1^ s^–1^ for their reactions with *t*BuO^•^ and tBuOO^•^, respectively, see ref (25).), points toward a negligible contribution for this pathway. We thank the reviewer for drawing our attention on this point.

[ref25] Warren J. J.; Mayer J. A. Predicting organic hydrogen atom transfer rate constants using the Marcus cross relation. Proc. Natl. Acad. Sci. U.S.A. 2010, 107, 5282–5287. 10.1073/pnas.0910347107. 20215463 PMC2851756

[ref26] aBernasconi C. F. The principle of nonperfect synchronization: recent developments. Adv. Phys. Org. Chem. 2010, 44, 223–324. 10.1016/S0065-3160(08)44005-4.

[ref27] Liu F.; Yang Z.; Yu Y.; Mei Y.; Houk K. N. Bimodal Evans–Polanyi Relationships in Dioxirane Oxidations of sp^3^ C–H: Non-perfect Synchronization in Generation of Delocalized Radical Intermediates. J. Am. Chem. Soc. 2017, 139, 16650–16656. 10.1021/jacs.7b07988. 29069541

[ref28] Salamone M.; Ortega V. B.; Bietti M. Enhanced reactivity in hydrogen atom transfer from tertiary sites of cyclohexanes and decalins via strain release: Equatorial C–H activation vs axial C–H deactivation. J. Org. Chem. 2015, 80, 4710–4715. 10.1021/acs.joc.5b00636. 25848679

[ref29] Maillard B.; Ingold K. U.; Scaiano J. C. Rate constants for the reactions of free radicals with oxygen in solution. J. Am. Chem. Soc. 1983, 105, 5095–5099. 10.1021/ja00353a039.

[ref30] Recupero F.; Punta C. Free Radical Functionalization of Organic Compounds Catalyzed by N-Hydroxyphthalimide. Chem. Rev. 2007, 107, 3800–3842. 10.1021/cr040170k. 17848093

[ref31] Yin H.; Xu L.; Porter N. A. Free radical lipid peroxidation: mechanisms and analysis. Chem. Rev. 2011, 111, 5944–5972. 10.1021/cr200084z. 21861450

[ref32] The experimental results support the hypothesis that this pathway is promoted by the methylperoxyl radical formed by oxygen trapping of the methyl radical deriving from competitive β-scission in *t*BuO^•^. See Schemes S3 and S4 and associated discussion in the SI.

[ref33] aHartung J.; Schneiders N.; Gottwald T. On the synthesis of β-bromohydrine ethers via intermolecular alkoxyl radical addition to bicyclo [2.2. 1] heptane. Tetrahedron Lett. 2007, 48, 6027–6030. 10.1016/j.tetlet.2007.06.103.

