# Supplementary material for: Hydrogen Atom Transfer-Based C(sp3)–H Bond Oxygenation of Lactams and Cycloalkenes: The Influence of Ring Size on Reactivity and Site Selectivity
Source: J Org Chem. 2025 Apr 7;90(15):5195–205. doi: 10.1021/acs.joc.5c00092 (PMC12012884; doi:10.1021/acs.joc.5c00092)
Supplement: Supplementary file 1 — jo5c00092_si_001.pdf [file jo5c00092_si_001.pdf]

Electronic Supporting Information

Hydrogen Atom Transfer based C(sp^3)–H Bond Oxygenation of Lactams and Cycloalkenes. The Influence of Ring Size on Reactivity and Site-selectivity.

Sergio Sisti,[a] Fabio Ioele,[a] Filippo Scarchilli,[a,b] Simona Laparelli,[a] Marco Galeotti,[a,b] Omid Hosseinzadeh,[c] Zhehan Jia,[c] Gino A. DiLabio,[c] Michela Salamone,*,[a] and Massimo Bietti *,[a]

[a] *Dipartimento di Scienze e Tecnologie Chimiche, Università "Tor Vergata", Via della Ricerca Scientifica, 1 I-00133 Rome, Italy.*

[b] *QBIS Research Group, Institut de Química Computacional i Catàlisi (IQCC) and Departament de Química, Universitat de Girona, Campus Montilivi, Girona E-17071, Catalonia, Spain.*

[c] *Department of Chemistry, The University of British Columbia, 3247 University Way, Kelowna, British Columbia, Canada, V1V 1V7*

1. Experimental Section **S2**

1.1 Instrumentation **S2**

1.2 Materials **S2**

1.2.1. Solvents

1.2.2. Reagents

1.2.3. Substrates

1.2.4. Reaction products

2. Synthesis of the substrates **S4**

3. Laser flash photolysis studies **S5**

3.1 k_{obs} vs [substrate] plots **S5**

4. Product studies **S9**

4.1 General procedure **S9**

4.2 Results **S10**

4.2.1 Mechanistic rationale for the formation of epoxidation and addition products **S19**

4.3 Characterization of the reaction products **S22**

4.3.1 Products from lactams **S22**

4.3.1.1 Products from *substrates* ***S1-S4***

4.3.1.2 Products from *N-methylpyrrolidine-2-one* ***(S5)***

4.3.1.3 Products from *N-methylpiperidin-2-one* ***(S6)***

4.3.1.4 Products from *N-methylazepan-2-one* ***(S7)***

4.3.1.5 Products from *N-methylazocan-2-one* ***(S8)***

4.3.1.6 Products from *N-benzylpyrrolidin-2-one* ***(S9)***

4.3.1.7 Products from *N-benzylpiperidin-2-one* ***(S10)***

4.3.1.8 Products from *N-benzylazepan-2-one* ***(S11)***

4.3.1.9 Products from *N-benzylazocan-2-one* ***(S12)***

4.3.2 Products from cycloalkenes **S28**

4.3.2.1 Products from *cyclopentene* ***(S13)***

4.3.2.2 Products from *cyclohexene* ***(S14)***

4.3.2.3 Products from *cycloheptene* ***(S15)***

4.3.2.4 Products from *cyclooctene* ***(S16)***

4.3.2.5 Products from *norbornene* ***(S17)***

4.3.2.6 Products from *styrene* ***(S18)***

5. NMR spectra **S30**

6. Computational studies **S42**

7. References **S44**

1. Experimental Section

1.1 Instrumentation

Laser flash photolysis (LFP) studies have been carried out using the third harmonic (355 nm) of a Q-switched Nd:YAG laser, delivering pulses of the duration of 8 ns. The laser energy has been adjusted by the use of the appropriate filter to ≤ 10 mJ/pulse. A 3.5 mL (or 0.5 mL) quartz cell (Suprasil, optical path 1 cm) has been used and all the experiments have been carried out at T = 25±0.5 °C under magnetic stirring.

Steady-state photolysis experiments were carried out employing a Helios Italquartz photoreactor, equipped with 10 × 15 W lamps with emission at λ_{max} = 310 nm. The reactions were carried out at constant temperature (T = 25 °C) by means of a Haake DC 10 thermostat linked to the outer casing of a Pyrex glass vessel containing the reaction mixture.

Gas-chromatographic analyses were carried out with a Agilent 7820A gas chromatograph equipped with a HP-5 30 m × 0.32 mm capillary column.

GC-MS analyses have been performed with a Shimadzu GC-MS QP2010 Ultra system.

^{1}H NMR and ^{13}C NMR spectra were recorded on a Bruker Avance 700 MHz spectrometer. Spectra are referenced to tetramethylsilane (TMS).

1.2 Materials

1.2.1. Solvents

Solvents used are of commercially available reagent quality unless stated otherwise. Spectroscopic grade acetonitrile (MeCN) has been employed in the LFP experiments.

1.2.2. Reagents

Commercially available dicumyl peroxide (≥ 98 %) has been used without further purification in all the time-resolved kinetic studies. Commercially available di-*tert*-butyl peroxide (≥ 98 %) has been used without further purification in all the steady-state photolysis studies.

All reagents used for synthetic purposes are commercially available and used as received.

1.2.3. Substrates

The structures of substrates **S1-S16** are displayed in Chart 1, accompanied by norbornene (**S17**) and styrene (**S18**) that have been employed as mechanistic probes.

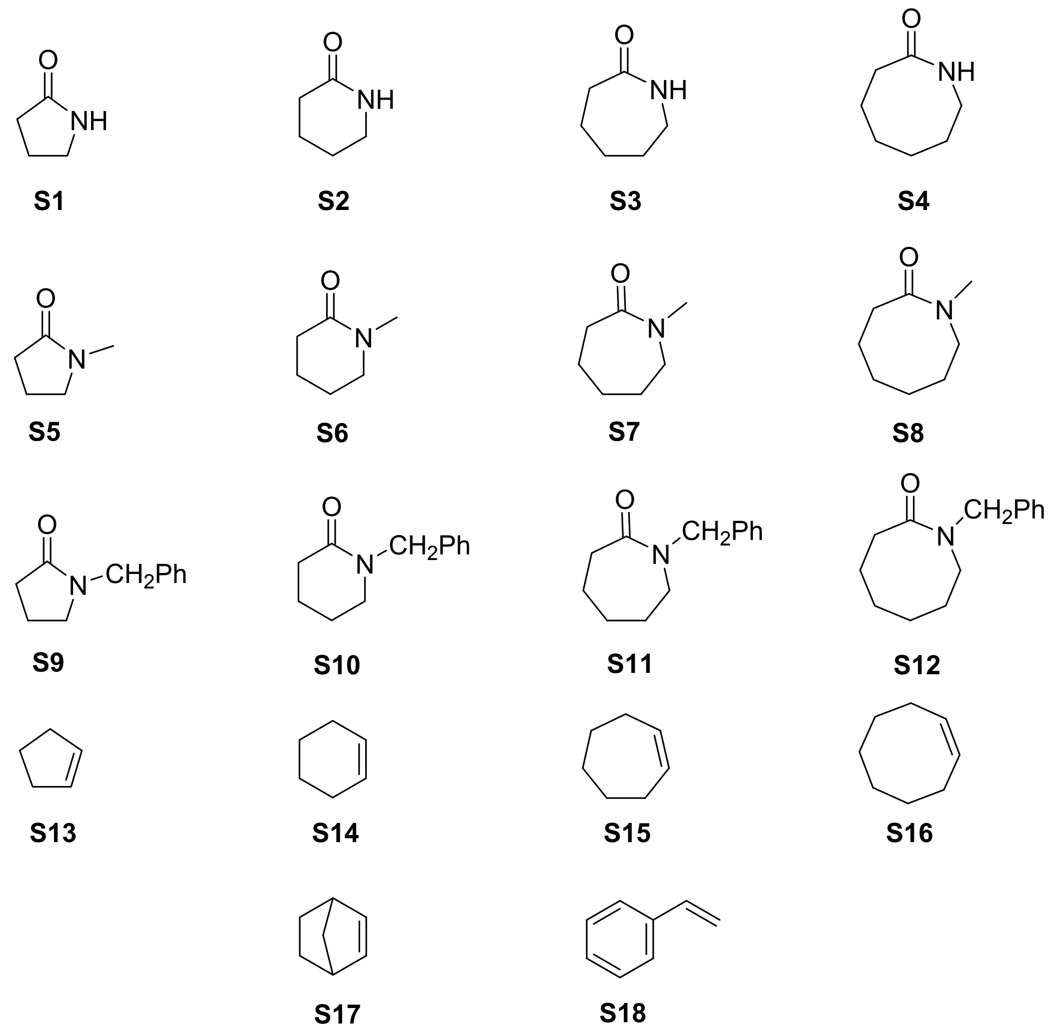

Chart 1

Substrates **S1-S7, S9** and **S13-S16** are commercially available.

Synthesis, isolation and characterization of *N*-methylazocan-2-one (**S8**), *N*-benzylpiperidin-2-one (**S10**), *N*-benzylazepan-2-one (**S11**) and *N*-benzylazocan-2-one (**S12**) are described below in paragraph **2**.

1.2.4. Reaction products

Products derived from the oxidation reactions have been identified by comparison with authentic samples or isolated from the reaction mixtures and identified by NMR and GC-MS analysis as reported in paragraph **4.3**. In some cases, because of the presence in the reaction mixture of several isomeric products formed in relatively low amount, the NMR spectra of the isolated products or product couples show the presence of non negligible amounts of impurities.

2. Synthesis of the substrates

Substrate **S8** has been synthesized by reacting azocan-2-one with methyl iodide in the presence of NaH in anhydrous THF under a nitrogen atmosphere following a previously reported procedure (**Scheme S1**).[S1] The crude has been purified by column chromatography on silica gel (eluent: CH_2Cl_2:MeOH 50:1) leading to a white solid with 88% yield (> 99% purity by GC).

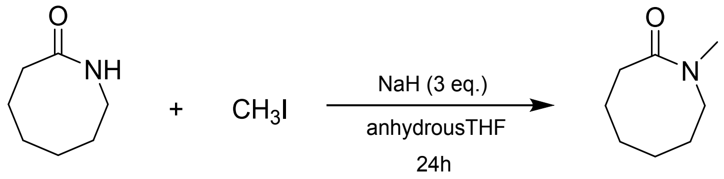

Scheme S1

^{1}H NMR (700 MHz, $CDCl_3$) δ 3.47 (t, *J* = 6.1 Hz, 2H), 2.90 (s, 3H), 2.49 (dd, *J* = 8.1, 4.7 Hz, 2H), 1.79 (m, 2H), 1.67-1.64 (m, 2H), 1.54-1.52 (m, 2H), 1.44-1.42 (m, 2H).

Substrates **S10**-**S12** have been synthesized by reacting the pertinent secondary lactam (2-piperidone (**S2**), azepan-2-one (**S3**) and azocan-2-one (**S4**)) with benzyl bromide in the presence of NaH in anhydrous THF under a nitrogen atmosphere according to a procedure reported in the literature (**Scheme S2).**[S2] The reaction products have been purified by flash chromatography (silica gel, eluent: pentane:diethyl ether, 76-95% yield).

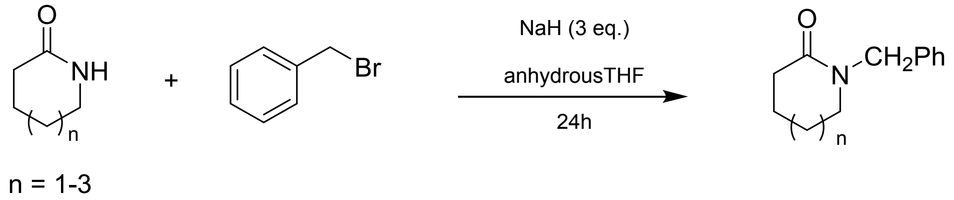

Scheme S2

N-benzylpiperidin-2-one (**S10**):[S3]

^{1}H NMR (700 MHz, $CDCl_3$): δ 7.32 - 7.25 (m, 5H), 4.6 (s, 2H), 3.23 (t, *J* = 5.9 Hz, 2H), 2.50 (t, *J* = 6.5 Hz, 2H), 1.8 – 1.77 (m, 4H).

N-benzylazepan-2-one (**S11**):[S4]

^{1}H NMR (700 MHz, $CDCl_3$): δ 7.31-7.25 (m, 5H), 4.59 (s, 2H), 3.30-3.28 (m, 2H), 2.61-2.60 (m, 2H), 1.71-1.70 (m, 4H), 1.50-1.49 (m, 2H).

N-benzylazocan-2-one (**S12**):

^{1}H NMR (700 MHz, $CDCl_3$): δ 7.28 (m, 5H), 4.58 (s, 2H), 3.39 (t, *J* = 5.6 Hz, 2H), 2.57 (m, 2H), 1.84 (m, 2H), 1.56 – 1.48 (m, 6H).

^{13}C {^{1}H} NMR (700 MHz, $CDCl_3$): δ 175.1, 137.9, 128.66, 128.03, 127.52, 47.7, 45.9, 33.9, 28.95, 28.62, 26.1, 26.0, 24.2.

3. Laser flash photolysis studies

Experiments have been typically carried out employing argon or nitrogen saturated acetonitrile solutions containing 1.0 M dicumyl peroxide. The observed rate constants (k_{obs}) have been obtained following the decay of the cumyloxyl radical (CumO$^{•}$) visible absorption band at 490 nm as a function of the concentration of added substrate[S5]. Second order rate constant (k_H) for the reactions of CumO$^{•}$ with substrates **S10-S16** have been obtained from the slopes of the k_{obs} vs [substrate] plots. The concentration variation was performed following two different approaches: dilution of a MeCN solution containing 1.0 M dicumyl peroxide and the substrate at the highest concentration employed, with a 1.0 M solution of dicumyl peroxide in MeCN (substrates **S10-S12**), or direct addition of the substrate to a 1.0 M solution of dicumyl peroxide in MeCN (substrates **S13-S16**). The k_H values are the average at least two values obtained through independent experiments, with typical errors being ≤ 10 %.

3.1 k_{obs} vs [substrate] plots

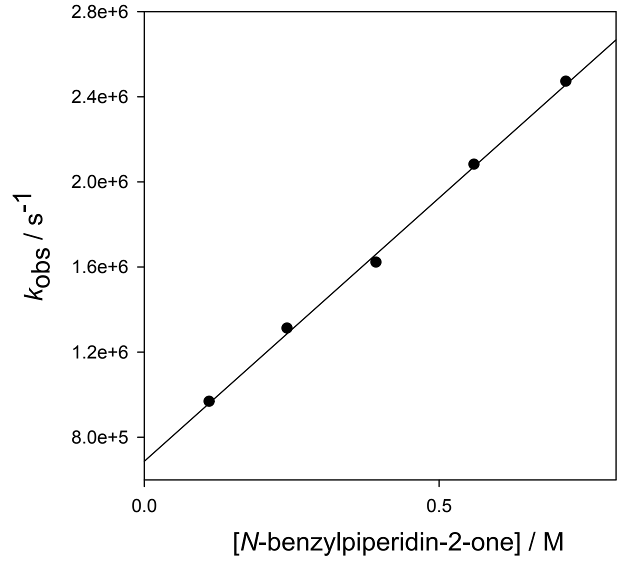

Figure S1. Plot of the observed rate constant (k_{obs}) against [substrate] for the reaction of *N*-benzylpiperidin-2-one (**S10**) with CumO$^{•}$ generated by 355 nm LFP of an Ar-saturated MeCN solution containing 1.0 M dicumyl peroxide measured at T = 25 °C following the decay of CumO$^{•}$ at 490 nm. From the linear regression analysis: intercept = 6.87×10^5 s^{-1}, $k_H = 2.48 \times 10^6$ M^{-1} s^{-1}, $r^2 = 0.998$. A duplicate of the experiment : intercept = 7.20×10^5 s^{-1}, $k_H = 2.51 \times 10^6$ M^{-1} s^{-1}, $r^2 =$ 0.997.
Average value: $\mathbf{k_H = (2.50 \pm 0.02) \times 10^6}$ **M^{-1} s^{-1}**.

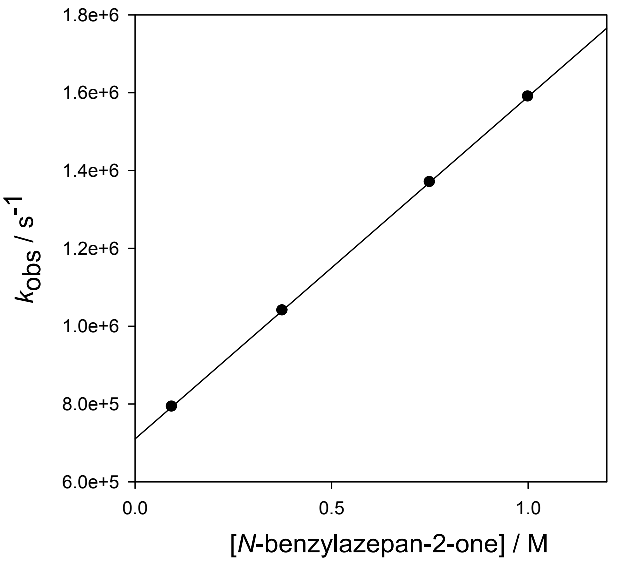

Figure S2. Plot of the observed rate constant (k_{obs}) against [substrate] for the reaction of *N*-benzylazepan-2-one (**S11**) with CumO$^•$ generated by 355 nm LFP of an Ar-saturated MeCN solution containing 1.0 M dicumyl peroxide measured at T = 25 °C following the decay of CumO$^•$ at 490 nm. From the linear regression analysis: intercept = 7.10 × 10^5 s^{-1}, k_H = 8.78 × 10^5 M^{-1} s^{-1}, r^2 = 0.999. A duplicate of the experiment : intercept = 7.53 × 10^5 s^{-1}, k_H = 9.01 × 10^5 M^{-1} s^{-1}, r^2 = 0.990.
Average value: $\boldsymbol{k_H}$ **= (8.9±0.1) × 10^5 M^{-1} s^{-1}**.

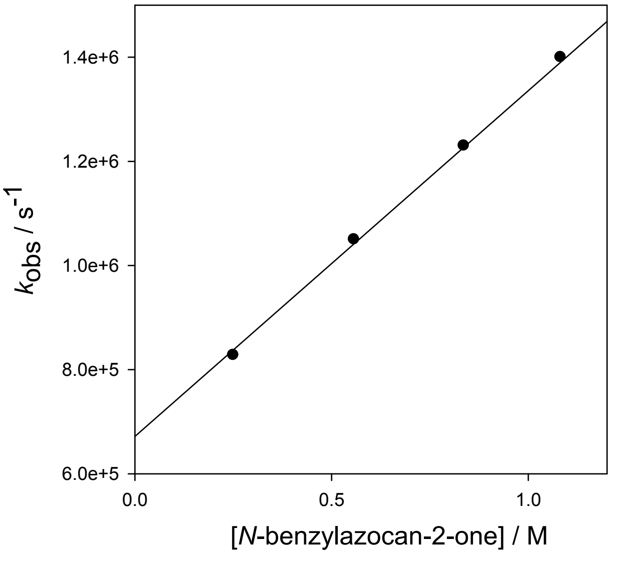

Figure S3. Plot of the observed rate constant (k_{obs}) against [substrate] for the reaction of *N*-benzylazocan-2-one (**S12**) with CumO$^•$ generated by 355 nm LFP of an Ar-saturated MeCN solution containing 1.0 M dicumyl peroxide measured at T = 25 °C following the decay of CumO$^•$ at 490 nm. From the linear regression analysis: intercept = 6.72 × 10^5 s^{-1}, k_H = 6.64 × 10^5 M^{-1} s^{-1}, r^2 = 0.999. A duplicate of the experiment : intercept = 7.12 × 10^5 s^{-1}, k_H = 6.36 × 10^5 M^{-1} s^{-1}, r^2 = 0.993.
Average value: $\boldsymbol{k_H}$ **= (6.5±0.1) × 10^5 M^{-1} s^{-1}**.

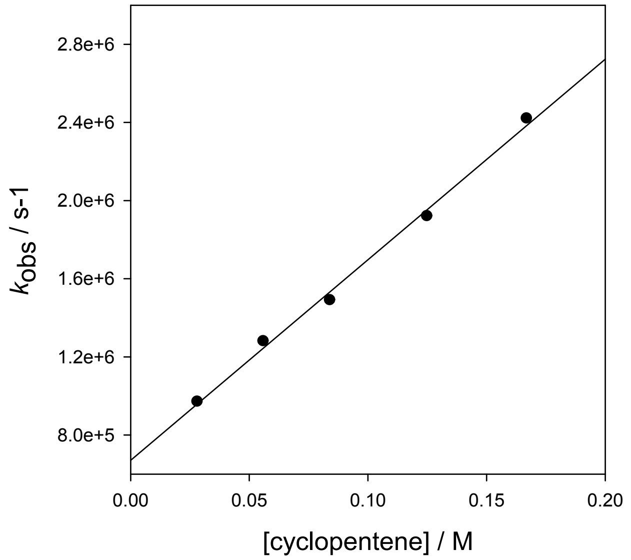

Figure S4. Plot of the observed rate constant (k_{obs}) against [substrate] for the reaction of cyclopentene (**S13**) with CumO$^{\bullet}$ generated by 355 nm LFP of an Ar-saturated MeCN solution containing 1.0 M dicumyl peroxide measured at T = 25 °C following the decay of CumO$^{\bullet}$ at 490 nm. From the linear regression analysis: intercept = 6.71 × 10^5 s^{-1}, k_H = 1.03 × 10^7 M^{-1} s^{-1}, r^2 = 0.996. A duplicate of the experiment : intercept = 6.88 × 10^5 s^{-1}, k_H = 9.63 × 10^6 M^{-1} s^{-1}, r^2 = 0.996. Average value: $\boldsymbol{k_H}$ **= (1.00±0.03) × 10^7 M^{-1} s^{-1}**.

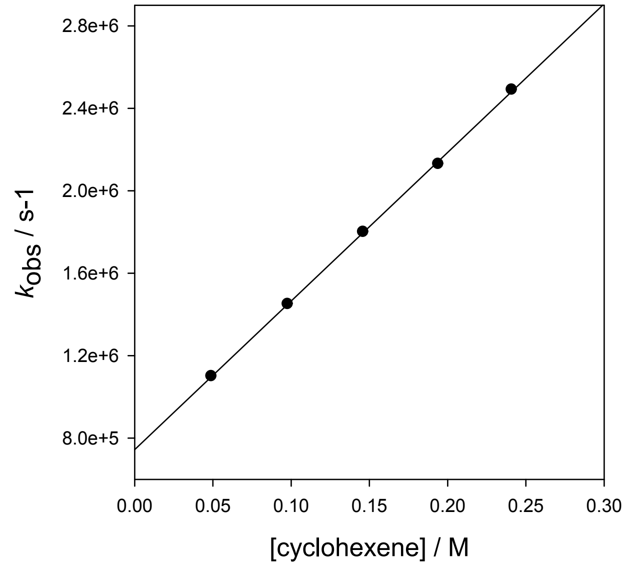

Figure S5. Plot of the observed rate constant (k_{obs}) against [substrate] for the reaction of cyclohexene (**S14**) with CumO$^{\bullet}$ generated by 355 nm LFP of an Ar-saturated MeCN solution containing 1.0 M dicumyl peroxide measured at T = 25 °C following the decay of CumO$^{\bullet}$ at 490 nm. From the linear regression analysis: intercept = 7.45 × 10^5 s^{-1}, k_H = 7.21 × 10^6 M^{-1} s^{-1}, r^2 = 0.999. A duplicate of the experiment : intercept = 7.33 × 10^5 s^{-1}, k_H = 7.11 × 10^6 M^{-1} s^{-1}, r^2 = 0.998. Average value: $\boldsymbol{k_H}$ **= (7.16±0.05) × 10^6 M^{-1} s^{-1}**.

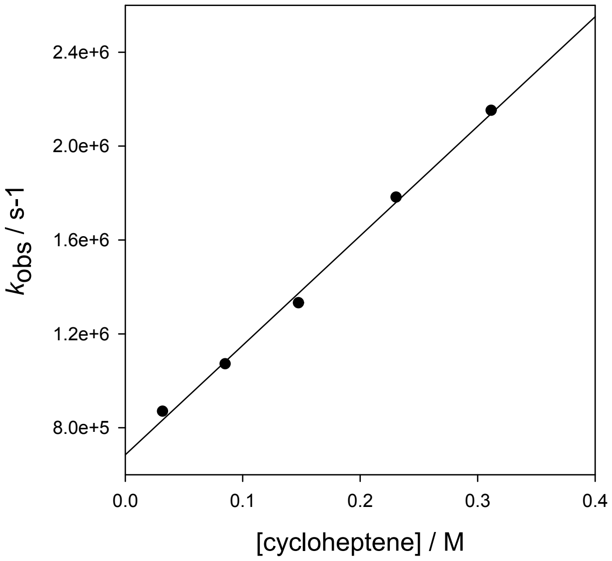

Figure S6. Plot of the observed rate constant (k_{obs}) against [substrate] for the reaction of cycloheptene (**S15**) with CumO• generated by 355 nm LFP of an Ar-saturated MeCN solution containing 1.0 M dicumyl peroxide measured at T = 25 °C following the decay of CumO• at 490 nm. From the linear regression analysis: intercept = 6.85×10^5 s^{-1}, $k_H = 4.67 \times 10^6$ M^{-1} s^{-1}, r^2 = 0.997. A duplicate of the experiment : intercept = 7.55×10^5 s^{-1}, $k_H = 4.53 \times 10^6$ M^{-1} s^{-1}, r^2 = 0.997. Average value: $\boldsymbol{k_H}$ **= (4.60±0.07) × 10^6 M^{-1} s^{-1}**.

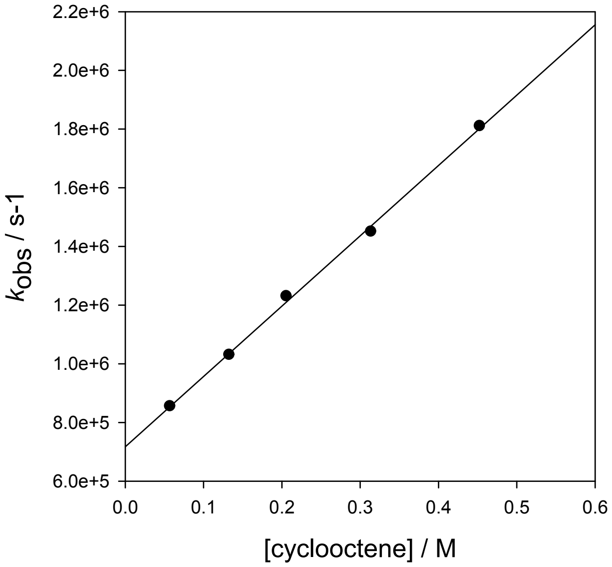

Figure S7. Plot of the observed rate constant (k_{obs}) against [substrate] for the reaction of cyclooctene (**S16**) with CumO• generated by 355 nm LFP of an Ar-saturated MeCN solution containing 1.0 M dicumyl peroxide measured at T = 25 °C following the decay of CumO• at 490 nm. From the linear regression analysis: intercept = 7.17×10^5 s^{-1}, $k_H = 2.40 \times 10^6$ M^{-1} s^{-1}, r^2 = 0.999. A duplicate of the experiment : intercept = 7.39×10^5 s^{-1}, $k_H = 2.31 \times 10^6$ M^{-1} s^{-1}, r^2 = 0.997. Average value: $\boldsymbol{k_H}$ **= (2.36±0.05) × 10^6 M^{-1} s^{-1}**.

4. Product studies

Product analysis of the reactions of the *tert*-butoxyl radical (*t*BuO$^•$) with substrates **S1**-**S16** have been carried out by means of 310 nm steady-state photolysis of O_2-saturated MeCN solutions containing 0.3-0.5 M di-*tert*-butyl peroxide (DTBP) and the hydrogen atom donor substrate (0.1-0.2 M), following the procedure reported in **paragraph 4.1**.

4.1 General procedure

A 5 ml solution of the substrate (0.1 M < [substrate] < 0.2 M) and di-*tert*-butyl peroxide (0.3 M < [di-*tert*-butyl peroxide] < 0.5 M) in MeCN has been introduced into a jacketed glass or quartz tube. The solution has been carefully saturated with oxygen and irradiated with 10×15 W UV lamps (emission maximum at 310 nm) for times between 3 and 9 hours (substrates **S1**-**S12**) or 15-120 minutes (substrates **S13**-**S16**) under stirring at T = 25 °C. The internal standard (*N,N*-dimethylacetamide, *N*-ethylpyrrolidin-2-one, azepan-2-one, cyclohexene, cyclooctene or toluene) has been then added and the solution directly analyzed without any workup. GC analysis of the solution afforded the conversions of the substrate and product yields relative to the internal standard integration. Oxidation products have been identified by comparison with authentic samples, commercially available or independently synthesized and previously identified by ^{1}H-NMR, ^{13}C NMR and GC-MS. Alternatively, products from different photo-oxidations were accumulated, isolated by column chromatography and characterized by NMR spectroscopy and/or GC-MS.

4.2 Results

The results obtained in the oxidation of substrates **S1-S16** promoted by the *t*BuO•/O_2 system are reported herein. Substrate conversions and product yields shown in **Tables S1-S13** were determined by GC analysis and represent the average of at least two independent experiments.

In order to verify that site-selectivity is unaffected by the nature of the abstracting *tert*-alkoxyl radical, the results obtained in the reaction of *t*BuO• with 1-methylazocan-2-one (**S7**) and cyclooctene (**S16**), were compared to those obtained for the corresponding reactions promoted by CumO•. Both substrates display almost identical product distributions in their reactions with the two radicals (see Table S4 and Table S13, respectively).

The results obtained in the oxidation of **norbornene** (**S17**) and **styrene** (**S18**) under analogous reaction conditions are also included and detailed in **Table S14** and **Table S15**, respectively.

Substrates S1-S4

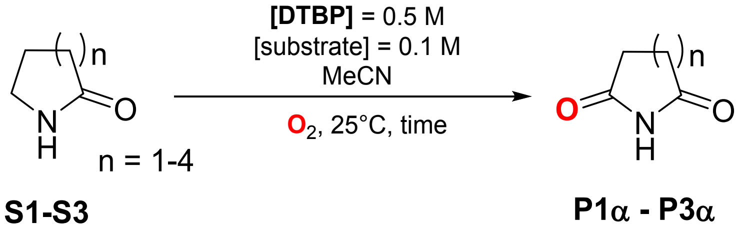

Table S1a. Results obtained from the aerobic oxidation of **S1-S3** promoted by *t*BuO•.[a]

Substrate (Sn)	*Time (h)*	*Conversion (%)*	*P1α-P3α (%)*
**S1**	3	75.5	69.5
	3[b]	55.0	48.0
	6	90.0	74.0
**S2**	3	96	89
**S3**	3	30.5	21.5

[a]Internal standard: *N,N*-dimethylacetamide. [b][**S1**] = 0.2 M.

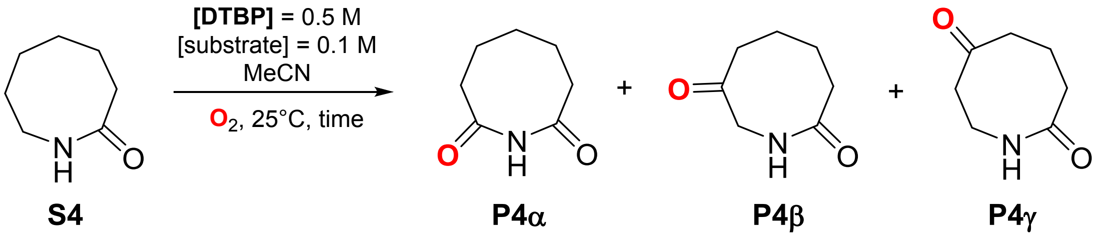

Table S1b. Results obtained from the aerobic oxidation of **S4** promoted by *t*BuO•.[a]

Substrate (Sn)	***Time (h)***	***Conversion (%)***	***P4α (%)***	***P4β (%)***	***P4γ (%)***	***Total Yield (%)***
**S4**	6	9.0	2.0	2.3	2.7	7.0
	9	15.5	3.0	3.2	3.8	10.0
	18	21.0	4.0	4.3	4.7	13.0

[a]Internal standard: azepan-2-one.

1-methylpyrrolidin-2-one (S5)

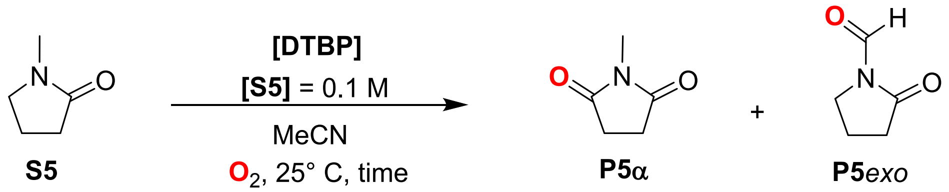

Table S2. Results obtained from the aerobic oxidation of **S5** promoted by *t*BuO•.[a]

[DTBP]/M	*Time (h)*	*Conversion (%)*	*P5α (%)*	*P5exo (%)*	*Total Yield (%)*
0.5	1	38	19	3	22
0.3	1	38	18	3	21
0.3	3	64	37	6	43

[a]Internal standard: *N,N*-dimethylacetamide

1-methylpiperidin-2-one (S6)

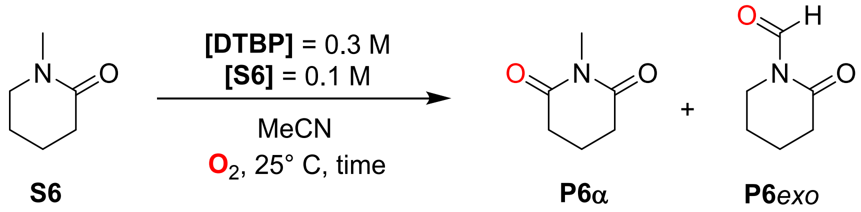

Table S3. Results obtained from the aerobic oxidation of **S6** promoted by *t*BuO•.[a]

Time (h)	*Conversion (%)*	*P6α (%)*	*P6exo (%)*	*Total Yield (%)*
1	30	10	2	12
3	57	41	7	48

[a]Internal standard: *N,N*-dimethylacetamide

N-methylazepan-2-one (S7)

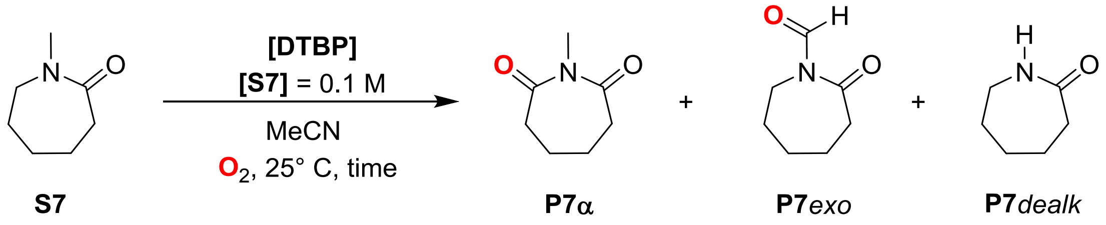

Table S4. Results obtained from the aerobic oxidation of **S7** promoted by *t*BuO•.[a]

[DTBP]/M	*Time (h)*	*Conversion (%)*	*P7α (%)*	*P7exo (%)*	*P7dealk (%)*	*Total Yield (%)*
0.3	3	34	10	18	5	31
0.3	6	58	12	23	8	46
0.5	6	75	11	21	7	43
0.3[b]	6	50	12	27	4	43

[a]Internal standard: *N,N*-dimethylacetamide. [b]Reaction promoted by CumO• generated by 310 nm photolysis of dicumyl peroxide under analogous experimental conditions.

N-methylazocan-2-one (S8)

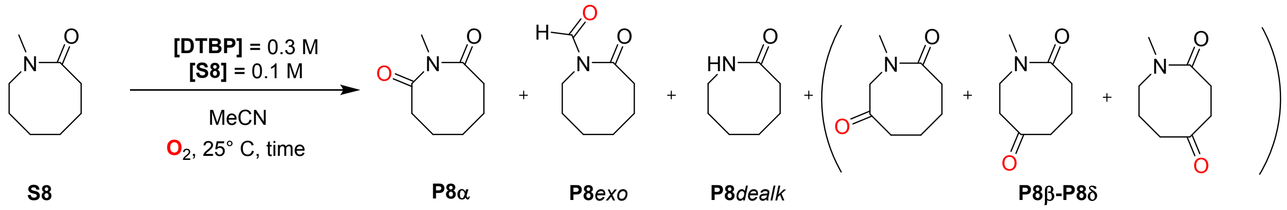

Table S5. Results obtained from the aerobic oxidation of **S8** promoted by *t*BuO•.[a]

Time (h)	*Conversion (%)*	*P8α (%)*	*P8exo (%)*	*P8dealk (%)*	*P8β-P8δ (%)*[b]	*Total Yield (%)*
3	31	1	7	6	6	20
6	47	1	9	9	9	28

[a]Internal standard: *N,N*-dimethylacetamide. [b]The given yield represents the sum of the three isomeric ketolactam products.

N-benzylpyrrolidin-2-one (S9)

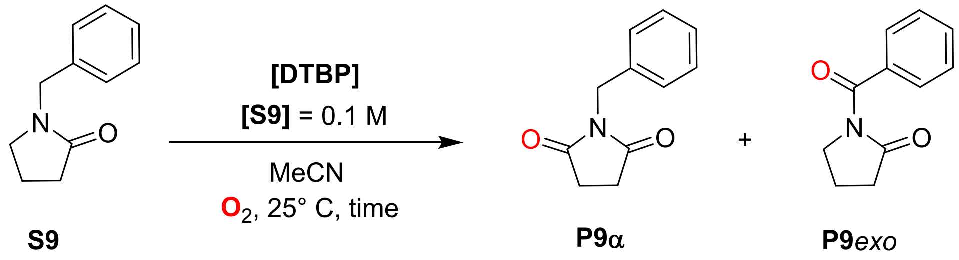

Table S6. Results obtained from the aerobic oxidation of **S9** promoted by *t*BuO•.[a]

[DTBP]/M	*Time (h)*	*Conversion (%)*	*P9α (%)*	*P9exo (%)*	*Total Yield (%)*
0.3	3	28	11	7	18
0.5	3	35	17	9	26

[a]Internal standard: *N*-ethylpyrrolidin-2-one.

N-benzylpiperidin-2-one (S10)

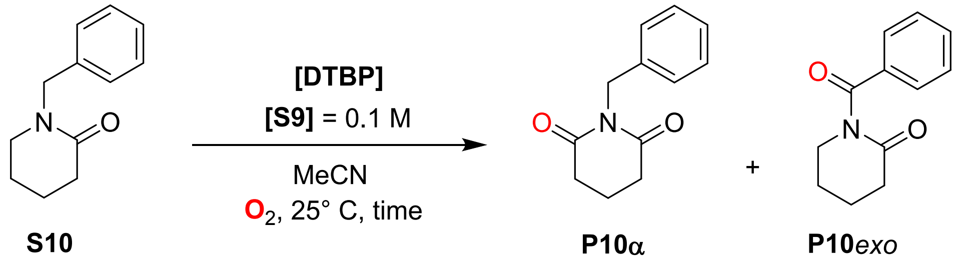

Table S7. Results obtained from the aerobic oxidation of **S10** promoted by *t*BuO•.[a]

[DTBP]/M	*Time (h)*	*Conversion (%)*	*P10α (%)*	*P10exo (%)*	*Total Yield (%)*
0.3	3	26	14	5	19
0.5	3	25	17	6	23
0.3	6	37	20	7	27
0.3	8	64	23	9	32

[a]Internal standard: *N*-ethylpyrrolidin-2-one.

N-benzylazepan-2-one (S11)

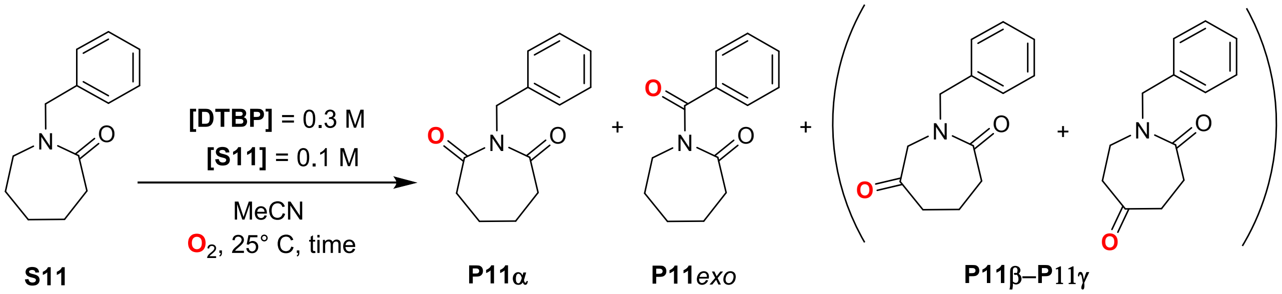

Table S8. Results obtained from the aerobic oxidation of **S11** promoted by *t*BuO•.[a]

Time (h)	*Conversion (%)*	*P11α (%)*	*P11exo (%)*	*P11β-P11γ (%)*[b]	*Total Yield (%)*
3	26	5	11	3	19
6	29	7	14	4	25
8	33	7	13	5	25

[a]Internal standard: *N,N*-dimethylacetamide. [b]The given yield represents the sum of the two isomeric ketolactam products.

N-benzylazocan-2-one (S12)

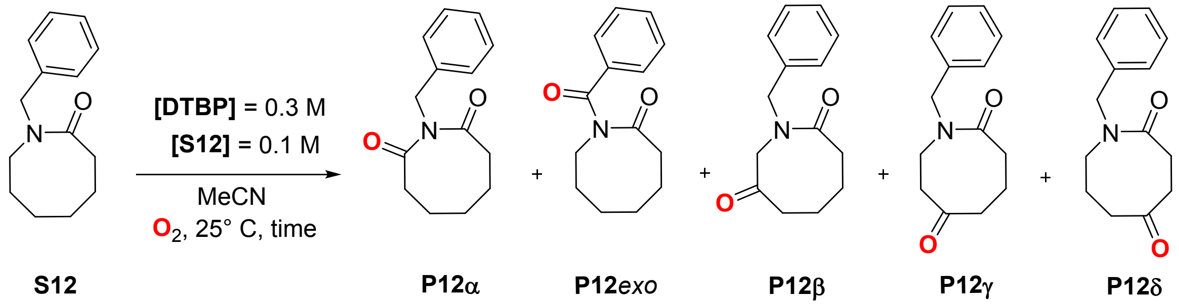

Table S9. Results obtained from the aerobic oxidation of **S12** promoted by *t*BuO•.[a]

time (h)	*Conversion (%)*	*P12α (%)*	*P12exo (%)*	*P12β (%)*	*P12γ (%)*	*P12δ (%)*	*Total Yield (%)*
3	16	0.6	4.6	4.4	1.9	3.7	16
6	32	1	7	6.2	2.6	5.2	22

[a]Internal standard: *N,N*-dimethylacetamide.

Cyclopentene (S13)

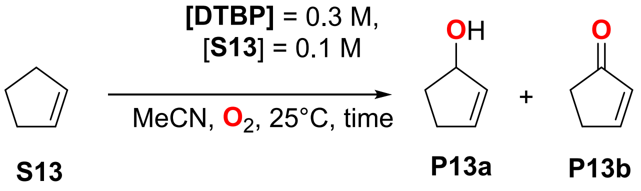

Table S10. Results obtained from the aerobic oxidation of **S13** promoted by *t*BuO•.[a]

Time (min)	*Conversion[b] (%)*	*P13a (%)*	*P13b (%)*	*Total Yield (%)*
15	-	1.8	2.8	4.6
30	-	3.9	6.0	9.9
60	-	5.1	11.5	16.6

[a]Internal standard: cyclooctene. [b]Substrate volatility prevented determination of the recovered substrate

Cyclohexene (S14)

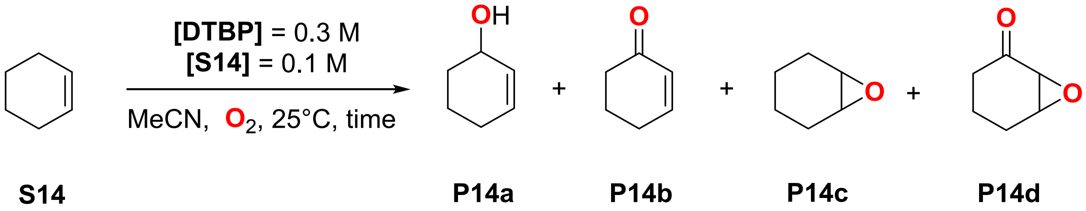

Table S11. Results obtained from the aerobic oxidation of **S14** promoted by *t*BuO•[a]

Time (min)	*Conversion (%)*	*P14a (%)*	*P14b (%)*	*P14c (%)*	*P14d (%)*	*Total Yield (%)*
5[b]	10.0	3.0	5.1	0.5	1.2	9.8
15[b]	21.0	7.3	8.7	0.9	1.0	17.9
30	38.5	6.6	10.5	1.7	1.4	20.2
30[c]	6.0	-	-	-	-	-

[a]Internal standard: cyclooctene. [b]For the definition of the relative importance of the competitive reaction pathways displayed in Figure 2b, products ratios obtained at 5 and 15 minutes have been employed, where, on the basis of the results displayed in Scheme S3 below, product **P14d** has been included among the products deriving from the HAT pathway. [c]Reaction carried out in the absence of DTBP.

Cycloheptene (S15)

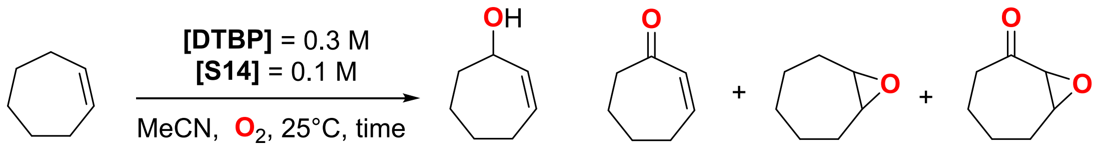

S15 **P15a** **P15b** **P15c** **P15d**

Table S12. Results obtained from the aerobic oxidation of **S15** promoted by *t*BuO•[a]

Time (min)	*Conversion (%)*	*P15a (%)*	*P15b (%)*	*P15c (%)*	*P15d (%)*	*Total Yield (%)*
15[b]	18.3	2.5	6.0	5.9	1.2	15.6
30	29.5	3.3	6.6	6.5	1.4	17.8
120	83.0	-	3.2	25.0	3.9	32.1

[a]Internal standard: cyclohexene. [b]For the definition of the relative importance of the competitive reaction pathways displayed in Figure 2b, products ratios obtained at 15 minutes have been employed, and product **P15d** has been included among the products deriving from the HAT pathway.

Cyclooctene (S16)

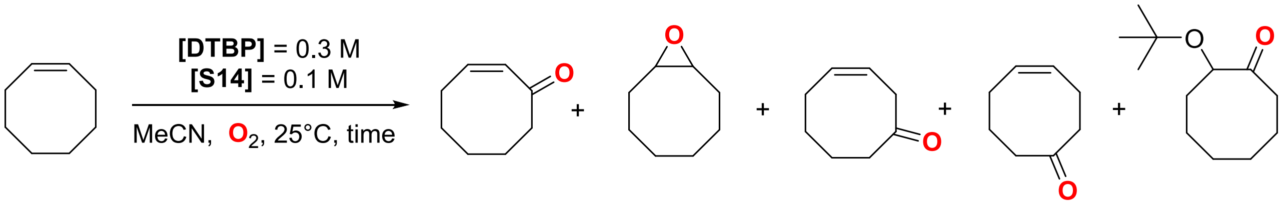

S16 **P16b** **P16c** **P16e** **P16f** **P16g**

Table S13. Results obtained from the aerobic oxidation of **S16** promoted by *t*BuO•.[a]

Time (min)	*Conversion (%)*	*P16b (%)*	*P16c (%)*	*P16e (%)*	*P16f (%)*	*P16g (%)*	*Total Yield (%)*
15[b]	11.5	1.7	4.6	0.1	3.0	1.8	11.2
30	26.0	2.3	8.9	0.2	4.5	2.7	18.6
30[c]	5	-	-	-	-	-	-
30[d]	23	2.4	8.8	0.2	4.5	2.8[e]	18.7

[a]Internal standard: cyclohexene. [b]For the definition of the relative importance of the competitive reaction pathways displayed in Figure 2b, products ratios obtained at 15 minutes have been employed. [c]Reaction carried out in the absence of DTBP. [d]Reaction promoted by CumO• generated by 310 nm photolysis of dicumyl peroxide under analogous experimental conditions. [e]Under these conditions the addition product **P16g** refers to 2-cumyloxy-cyclooctan-1-one

Norbornene (S17)

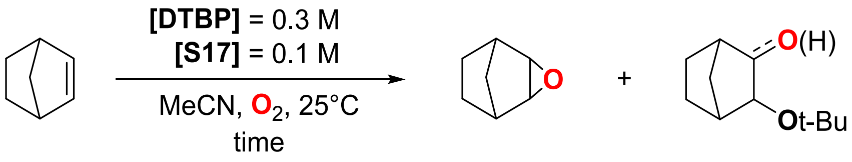

S17 P17c P17g

Table S14. Results obtained from the aerobic oxidation of **S12** promoted by *t*BuO•.[a]

Time (min)	*Conversion (%)*	*P17c(%)*	*P17g (%)*	*Total Yield (%)*
30	30.0	13.0	2.5	15.5
60	52.5	18.0	4.0	22.0
120	97.0	38.0	5.0	43.0
120[b]	49.0	-	-	-

[a]Internal standard: cyclooctene. [b]Reaction carried out in the absence of DTBP.

Styrene (S18)

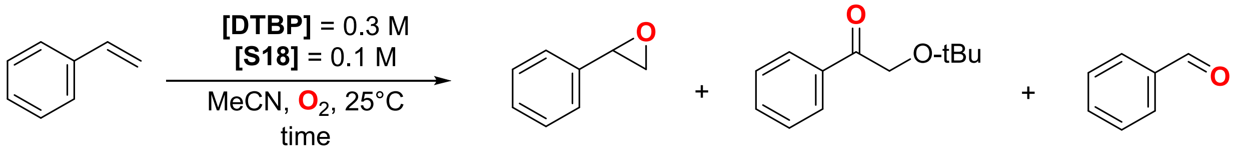

S18 P18c P18g P18h

Table S15. Results obtained from the aerobic oxidation of **S18** promoted by *t*BuO•.[a]

Time (h)	*Conversion (%)*	*P18c (%)*	*P18g (%)*	*P18h (%)*	*Other products (%)*	*Total Yield (%)*
1	35.2	6.5	4.6	12.4	5.8	29.3
2	52.5	15.6	5.0	14.2	7.7	42.5
6	91.0	29.4	4.8	18.9	8.5	61.6
2[b]	9.0	--	--	-	8.0	8.0

[a]Internal standard: toluene. [b]Reaction carried out in the absence of DTBP.

4.2.1 Mechanistic rationale for the formation of epoxidation and addition products

Formation of the epoxidation products can be explained on the basis of a previously proposed mechanism through addition of a peroxyl radical to the C=C double bond, followed by intramolecular homolytic substitution on the peroxide bond with release of an alkoxyl radical (Scheme S3), indicating that formation of these products does not contribute to the decay of CumO•.[S6]

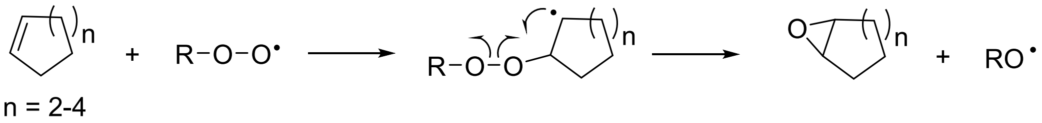

Scheme S3. Proposed mechanism for the formation of epoxidation products **P14c-P16c** in the reactions of cycloalkenes **S14-S16**.

The lack of epoxidation products in the reaction of **S13**, accompanied by the observation that the relative amount of these products increases with increasing ring-size (see Figure 2b) supports the hypothesis that this reactive pathway is promoted by the methylperoxyl radical (Scheme S3, R = CH_3) formed by oxygen trapping of the methyl radical deriving from competitive β-scission in *t*BuO• (Scheme S4). Because, as mentioned in the main text, the rate constant for the competitive bimolecular HAT reaction has been shown to decrease with increasing ring size, at constant substrate concentration, the relative importance of the unimolecular C–CH_3 β-scission pathway increases in the same direction, lending support to the intermediacy of the methylperoxyl radical in the epoxidation reaction.

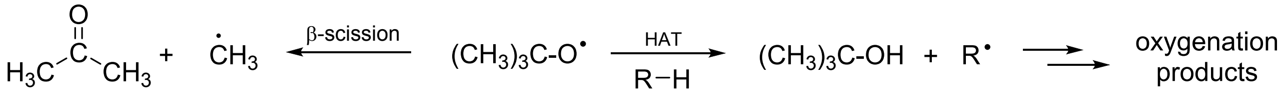

Scheme S4. Competitive reaction pathways for *t*BuO•.

In order to verify the origin of product **P14d**, we have carried out the following two experiments, subjecting ketone **P14b** and epoxide **P14c** to the reaction conditions (Scheme S5).

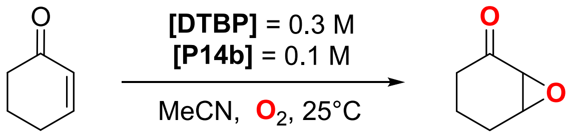

P14b **P14d**

time	conversion	yield
15 min.	5%	3%
30 min.	12%	10%

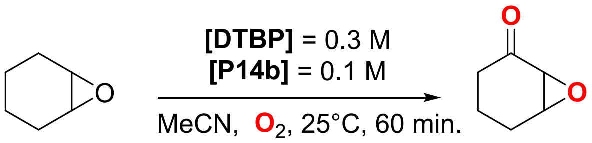

P14c **P14d**

> 99% rsm

Scheme S5

The observation that **P14b** led to the formation of **P14d**, whereas P14c was quantitatively recovered from the reaction mixture provides strong support to the hypothesis that **P14d** derives from epoxidation of the first formed **P14b**.

Formation of product **P16g**, can be rationalized in terms of the addition of *t*BuO• to the C=C bond of **S16**, followed by oxygen trapping (Scheme S6).

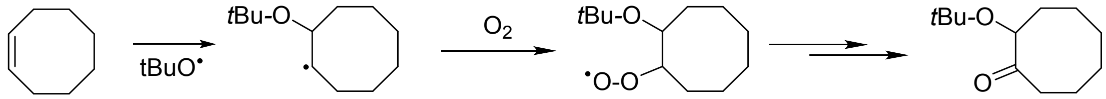

S16 **P16g**

Scheme S6. Proposed mechanism for the formation of 2-(*tert*-butoxy)-cyclooctanone (**P16g**) following addition of *t*BuO• to **S16**.

In order to provide support to the contribution of the epoxidation and *tert*-butoxyl addition pathways, norbornene (**S17**) and styrene (**S18**) were subjected to the same conditions employed for the oxygenation of **S13-S16**. The pertinent results are displayed above in Tables S14 and S15. Both substrates lack C–H bonds that are activated toward HAT to *tert*-alkoxyl radicals and are therefore customarily employed as probes for the study of radical C=C bond addition reactions.[S7]. With both **S17** and **S18** formation of the epoxidation (**P17c** and **P18c**) and *tert*-butoxyl addition products (**P17g** and **P18g**) was observed (Scheme S7) lending support to the mechanisms proposed in

Schemes S3 and S6. With **S18**, benzaldehyde (**P18h**) was also observed among the reaction products.

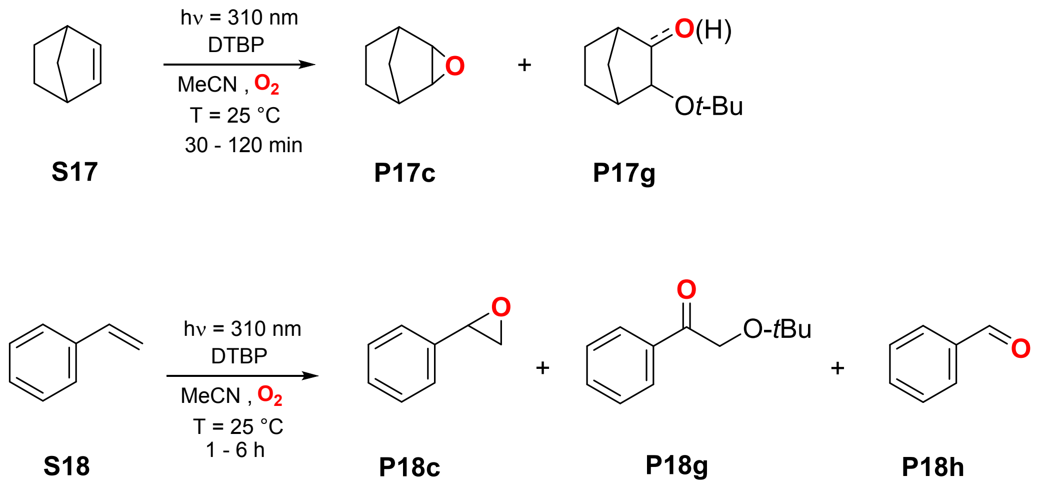

Scheme S7. Structure of the products observed in the oxygenation of **S17** and **S18** promoted by *t*BuO•.

4.3 Characterization of the reaction products

4.3.1 Products from lactams

4.3.1.1 Products from *substrates S1-S4*

<u>**Succinimide (P1α)**</u> has been identified by comparison with an authentic sample.

<u>**Glutarimide (P2α)**</u>

The crude obtained collecting two photoreactions carried out on 47 μL (0.5 mmol) of substrate **S2** and 460 μL (2.5 mmol) of DTBP has been purified by flash chromatography on silica gel (eluent: hexane/ethyl acetate 3:1) leading to 95 mg of a white solid (>99% purity by GC) identified as glutarimide (**P2α**) by GC-MS and ^{1}H NMR, according to spectroscopic data reported in the literature.[S8]

GC-MS (EI, 70 eV): m/z (%): 113 (35) [M^+], 70 (25), 42(100).

^{1}H NMR (700 MHz, $CDCl_3$): δ 7.84 (s, 1H); 2.60 (t, J = 6.5 Hz, 4H), 2.02 – 2.01 (m, 2H).

<u>**Azepan-2,7-dione (P3α)**</u>

The crude obtained collecting two photoreactions carried out on 57 μL (0.5 mmol) of substrate **S3** and 460 μL (2.5 mmol) of DTBP has been purified by flash chromatography on silica gel (eluent: hexane/ethyl acetate 2:1) leading to 23 mg of a white solid (>99% purity by GC) identified as azepan-2,7-dione (**P3α**) by GC-MS and ^{1}H NMR.[S9]

GC-MS (EI, 70 eV): m/z (%): 127 (30), 99 (18), 84 (23), 55 (100), 42 (18).

^{1}H NMR (700 MHz, $CDCl_3$): δ 7.73 (bs, 1H); 2.76 (bs, 4H), 1.96 (bs, 4H).

<u>**Azocan-2,n-dione**</u>

In order to identify the isomeric ketolactams, the crude obtained collecting two photoreactions carried out on 64 μL (0.5 mmol) of substrate **S4** and 460 μL (2.5 mmol) of DTBP has been subjected to benzylation following the procedure reported in paragraph 2. Three products were observed, and the chromatogram of the reaction was compared with that obtained from photoreaction of **S12,** the products of which have been unambiguously isolated and characterized (vide infra). The three have been identified as *N*-benzylazocane-2,8-dione (P12α), *N*-benzylazocane-2,7-dione (P12β), and *N*-benzylazocane-2,6-dione (P12γ), leading to the identification of the products obtained from the photoreaction of **S4** as azocan-2,8-dione (**P4α**), azocan-2,7-dione (**P4β**) and azocan-2,6-dione (**P4γ**).

Azocan-2,8-dione (P4α): GC-MS (EI, 70 eV): m/z (%): 141 (51), 113 (33), 99 (17), 85 (15), 64 (23), 59 (24), 55 (100), 42 (61), 30 (24)

Azocan-2,7-dione (P4β): GC-MS (EI, 70 eV) m/z (%): 141 (27), 139 (24), 100 (33), 84 (40), 69 (25), 54 (54), 42 (100), 30 (86).

Azocan-2,6-dione (P4γ): GC-MS (EI, 70 eV) m/z (%): 141 (6), 113 (100), 84 (40), 71 (10), 55 (52), 42 (86), 30 (44).

4.3.1.2 Products from *N-methylpyrrolidine-2-one (S5)*

***N*-methylsuccinimide (P5α)** has been identified by comparison with an authentic sample.

2-Oxopyrrolidine-1-carbaldehyde (P5*exo*)

The crude obtained collecting three photoreactions carried out on 48 μL (0.5 mmol) of substrate **S5** and 275 μL (1.5 mmol) of DTBP has been purified by flash chromatography on silica gel (eluent: hexane/ethyl acetate 1:1) leading to 8 mg of a colorless oil (> 99% purity by GC) identified by ^{1}H NMR, in line with spectroscopic data reported in the literature.[S10]

^{1}H NMR (700 MHz, $CDCl_3$): δ 9.10 (s, 1H), 3.76 (ddd, J = 7.8, 5.8, 2.1 Hz, 2H), 2.63 (dd, J = 14.0, 6.0 Hz, 2H), 2.19 – 2.09 (m, 2H).

4.3.1.3 Products from *N-methylpiperidin-2-one (S6)*

The crude obtained collecting two photoreactions carried out on 57 μL (0.5 mmol) of substrate **S6** and 275 μL (1.5 mmol) of DTBP has been purified by flash chromatography on silica gel (eluent: hexane/ethyl acetate 1:1) leading to 40 mg of a colorless oil (> 99% purity by GC) identified by ^{1}H-NMR and ^{13}C-NMR as *N*-methylpiperidine-2,6-dione (**P6α**) and 7 mg of a colorless oil (97% purity by GC) identified by ^{1}H-NMR as 2-oxopiperidine-1-carbaldehyde (**P6***exo*), in line with spectroscopic data reported in the literature.[S10]

***N*-methylpiperidine-2,6-dione (P6α)**

^{1}H NMR (700 MHz, $CDCl_3$): δ 3.14 (s, 3H), 2.67 (t, J = 6.5 Hz, 4H), 1.96-1.95 (m, 2H).

^{13}C {^{1}H} NMR (700 MHz, $CDCl_3$): δ 173.1, 32.9, 26.6, 17.4.

2-oxopiperidine-1-carbaldehyde (P6*exo*).

^{1}H NMR (700 MHz, $CDCl_3$): δ 8.09 (s, 1H), 3.33 (t, 2H), 2.39 (t, J = 6.5 Hz, 2H), 1.82 – 1.76 (m, 4H).

4.3.1.4 Products from *N-methylazepan-2-one (S7)*

The crude obtained collecting three photoreactions carried out on 65 μL (0.5 mmol) of substrate **S7** and 275 μL (1.5 mmol) of DTBP has been purified by flash chromatography on silica gel (eluent: hexane/ethyl acetate 2:1) leading to 15 mg of a colorless oil (> 99% purity by GC) identified by ^{1}H-NMR and ^{13}C-NMR as *N*-methylazepan-2,7-dione (**P7α**), 31 mg of a colorless oil (> 99% purity by GC) identified by ^{1}H-NMR and ^{13}C-NMR as 2-oxoazepan-1-carbaldehyde (**P7***exo*) and 8 mg of a colorless oil identified as azepan-2-one (**P7***dealk*) by comparison with an authentic sample.

***N*-methylazepan-2,7-dione (P7α)**

^{1}H NMR (700 MHz, $CDCl_3$): δ 3.16 (s, 3H), 2.80 (m, 4H), 1.89 (m, 4H).

^{13}C {^{1}H} NMR (700 MHz, $CDCl_3$): δ 176.1, 35.9, 30.1, 20.4.

2-oxoazepan-1-carbaldehyde (P7*exo*)

^{1}H NMR (700 MHz, $CDCl_3$): δ 9.39 (s, 1H), 3.80 (m, 4H), 2.69 (m, 2H), 1.80 – 1.68 (m, 6H).

^{13}C {^{1}H} NMR (700 MHz, $CDCl_3$): δ 171.9, 162.1, 40.1, 38.2, 29.5, 28.5, 23.4.

4.3.1.5 Products from *N-methylazocan-2-one (S8)*

The crude obtained collecting four photoreactions carried out on 78 μL (0.5 mmol) of substrate **S8** and 275 μL (1.5 mmol) of DTBP has been purified by flash chromatography on silica gel (eluent: hexane/ethyl acetate 2:1) leading to 50 mg of a colorless oil (> 99% purity by GC) identified by ^{1}H-NMR and ^{13}C-NMR as *N*-methylazocan-2,8-dione (**P8α**), 55 mg of a colorless oil (> 99% purity by GC) identified by ^{1}H-NMR and ^{13}C-NMR as 2-oxoazocan-1-carbaldehyde (**P8***exo*) and 5 mg of a colorless oil identified as azocan-2-one (**P8***dealk*) by comparison with an authentic sample.

***N*-methylazocan-2,8-dione (P8α)**

^{1}H NMR (700 MHz, $CDCl_3$): δ 3.23 (s, 3H), 3.06 (t, J = 6.1 Hz, 4H), 1.94 – 1.85 (m, 4H), 1.62 – 1.56 (m, 2H).

^{13}C {^{1}H} NMR (700 MHz, $CDCl_3$): δ 175.1, 38.1, 30.7, 24.05, 23.98.

2-oxoazocan-1-carbaldehyde (P8*exo*)

^{1}H NMR (700 MHz, $CDCl_3$): δ 9.45 (s, 1H), 3.86 (m, 2H), 2.67 (dd, J = 7.5, 5.4, 2H), 1.93 – 1.88 (m, 2H), 1.71 – 1.52 (m, 6H).

^{13}C {^{1}H} NMR (700 MHz, $CDCl_3$): δ 178.3, 162.6, 40.5, 35.0, 29.0, 28.6, 25.8, 24.3.

Moreover, from the same reaction mixture, fractions containing *N*-methylazocane-2,7-dione, *N*-methylazocane-2,5-dione and *N*-methylazocane-2,6-dione (products **P8β- P8δ**) have been obtained and the three isomers were identified by ^{1}H NMR.

***N*-methylazocan-2,7-dione (P8β)**: ^{1}H NMR (700 MHz, $CDCl_3$) δ 3.44 -3.43 (m, 2H), 2.93 (s, 2H), 2.73 (s, 3H), 2.47 (dd, *J* = 12.5, 6.1 Hz, 2H), 1.90 – 1.87 (m, 2H), 1.64 – 1.62 (m, 2H).

***N*-methylazocan-2,6-dione (P8γ)**: ^{1}H NMR (700 MHz, $CDCl_3$) δ 3.36 (dd, *J* = 11.6, 7.3 Hz, 2H 2H), 3.12 (s, 3H), ^{1}H NMR (700 MHz, $CDCl_3$) δ 2.68 (t, *J* = 7.2 Hz, 1H), 2.46 – 2.43 (m, 1H), 2.41 (dd, *J* = 13.8, 6.7 Hz, 1H),1.62 - 1.64 (m, 2H).

***N*-methylazocan-2,5-dione (P8δ)**: ^{1}H NMR (700 MHz, $CDCl_3$) δ 3.86 – 3.84 (m, 2H), 2.97 (s, 3H), 2.73 -2.71 (m, 2H), 2.63 -2.62 (m, 2H), 2.59 - 2.57 (dd, *J* = 15.1, 8.9 Hz, 2H), 1.98 - 1.95 (m, 2H).

4.3.1.6 Products from *N-benzylpyrrolidin-2-one (S9)*

***N*-benzylsuccinimide (P9α)** and ***N*-benzoylpyrrolidin-2-one (P9*exo*)** have been identified by comparison with authentic samples.

4.3.1.7 Products from *N-benzylpiperidin-2-one (S10)*

The crude obtained collecting two photoreactions carried out on 180 μL (1.0 mmol) of substrate **S10** and 275 μL (1.5 mmol) of DTBP has been purified by flash chromatography on silica gel (eluent: hexane/ethyl acetate 2:1) leading to 10 mg of a white solid (> 99% purity by GC) identified by ^{1}H NMR as *N*-benzylpiperidin-2,6-dione (**P10α**), in line with spectroscopic data reported in the literature.[S11]

***N*-benzylpiperidine-2,6-dione (P10α)**

^{1}H NMR (700 MHz, $CDCl_3$): δ 7.36 – 7.28 (m, 5H), 4.95 (s, 2H), 2.77 (t, *J* = 6.5 Hz, 4H), 1.95 (m, 2H).

***N*-benzoylpiperidine-2-one (P10*exo*)** has been identified by comparison with an authentic sample.

4.3.1.8 Products from *N-benzylazepan-2-one (S11)*

The crude obtained collecting four photoreactions carried out on 200 mg (1.0 mmol) of substrate **S11** and 275 μL (1.5 mmol) of DTBP has been purified by flash chromatography on silica gel (eluent: hexane/ethyl acetate 2:1) leading to 32 mg of a white solid (> 99% purity by GC) identified by ^{1}H NMR and ^{13}C NMR as *N*-benzoylazepan-2-one (**P11*exo***) and 100 mg of a solid containing *N*-benzoylazepan-2-one (**P11*exo***) and *N*-benzylazepan-2,7-dione (**P11α**) in a 1:1 mixture.

***N*-benzoylazepan-2-one (P11*exo*)**

^{1}H NMR (700 MHz, $CDCl_3$): δ 7.55-7.38 (m, 5H), 3.96 (bs, 2H), 2.71-2.70 (m, 2H), 1.88-1.84 (m, 6H).

^{13}C {^{1}H} NMR (700 MHz, $CDCl_3$): δ 177.5, 174.1, 136.5, 131.3, 128.1, 127.6, 45.1, 38.8, 29.57, 29.16, 23.7.

***N*-benzylazepan-2,7-dione (P11α)**

^{1}H NMR (700 MHz, $CDCl_3$): δ 7.32 (d, *J* = 7.5 Hz, 2H), 7.27 (t, *J* = 7.5 Hz 1H), 7.22 (t, *J* = 7.1 Hz, 2H), 4.92 (s, 2H), 2.78 (m, 4H), 1.83 (m, 4H).

***N*-benzylazepan-2,6-dione and *N*-benzylazepan-2,5-dione (P11β- P11γ)** were identified by GC-MS:

GC-MS (EI, 70 eV): m/z (%): 217 (20), 188 (17), 171 (29), 146 (14), 132 (29), 118 (20), 91 (100), 65 (22), 41 (12).

GC-MS (EI, 70 eV): m/z (%): 217 (68), 188 (2), 160 (17), 106 (39), 91 (100), 65 (18), 55 (12), 41(3).

4.3.1.9 Products from *N-benzylazocan-2-one (S12)*

N*-benzoylazocan-2-one** (**P12exo*) has been identified by comparison with an authentic sample.

***N*-benzylazocan-2,8-dione (P12α)**

The crude obtained collecting four photoreactions carried out on 200 μL (1.0 mmol) of substrate **S12** and 275 μL (1.5 mmol) of DTBP has been purified by flash chromatography on silica gel (eluent: hexane/ethyl acetate 2:1) leading to 110 mg of a white solid containing *N*-benzylazocane-2,8-dione (**P12α**) and *N*-benzoylazocane-2-one (**P12***exo*), with the former identified from this mixture by ^{1}H NMR.

^{1}H NMR (700 MHz, $CDCl_3$): δ 7.35-7.25 (m, 5H), 5.05 (s, 2H), 3.03 (m, 4H), 1.26 (m, 6H).

From the same column chromatography, the following products were also isolated (all white solids in > 99% GC purity): 20 mg of a product identified as *N*-benzylazocane-2,7-dione (**P12β**), 41 mg of a product identified as *N*-benzylazocane-2,6-dione (**P12γ**) and 34 mg of a product identified as *N*-benzylazocan-2,5-dione (**P12δ**).

***N*-benzylazocan-2,7-dione (P12β)**

^{1}H NMR (700 MHz, $CDCl_3$): δ 7.33 – 7.27 (m, 5H), 4.64 (s, 2H), 3.68 (s, 2H), 3.40 –3.37 (m, 2H), 2.58-2.54 (m, 2H), 1.87 – 1.68 (m, 4H).

^{13}C {^{1}H} NMR (700 MHz, $CDCl_3$): δ 205.6, 167.0, 136.7, 128.66, 128.20, 127.65, 50.8, 48.8, 46.4, 42.2, 26.6, 22.4.

***N*-benzylazocan-2,6-dione (P12γ)**

^{1}H NMR (700 MHz, $CDCl_3$): δ 7.35 – 7.27 (m, 5H), 4.64 (s, 2H), 3.71 – 3.67 (m, 2H), 2.64 – 2.56 (m, 6H), 2.04 – 2.01 (m, 2H).

^{13}C {^{1}H} NMR (700 MHz, $CDCl_3$): δ 211.4, 173.1, 136.9, 128.68, 128.25, 127.65, 48.0, 45.8, 43.2, 40.4, 33.6, 23.5.

***N*-benzylazocan-2,5-dione (P12δ)**

^{1}H NMR (700 MHz, $CDCl_3$): δ 7.34 – 7.26 (m, 5H), 4.58 (s, 2H), 3.38 – 3.34 (m, 2H), 2.85 – 2.82 (m, 4H), 2.48 – 2.44 (m, 2H), 1.82 – 1.74 (m, 2H).

^{13}C {^{1}H} NMR (700 MHz, $CDCl_3$): 212.2, 172.6, 136.9, 128.58, 128.23, 127.57, 48.4, 46.7, 44.7, 38.4, 30.25, 25.5.

4.3.2 Products from cycloalkenes

4.3.2.1 Products from *cyclopentene (S13)*

2-cyclopenten-1-ol (P13a) and **2-cyclopenten-1-one (P13b)** have been identified by comparison with authentic samples.

4.3.2.2 Products from *cyclohexene (S14)*

2-cyclohexen-1-ol (P14a) has been identified by comparison with an authentic sample.
2-cyclohexen-1-one (P14b) has been identified by comparison with an authentic sample.
cyclohexene oxide (P14c) has been identified by comparison with an authentic sample.
2-cyclohexen-1-one oxide (P14d) has been identified by comparison with an authentic sample.

4.3.2.3 Products from *cycloheptene (S15)*

2-cyclohepten-1-ol (P15a) has been identified by comparison with an authentic sample.
2-cyclohepten-1-one (P15b) has been identified by comparison with an authentic sample.
cycloheptene oxide (P15c) has been identified by comparison with an authentic sample.
2-cyclohepten-1-one oxide (P15d) has been identified by comparison with an authentic sample.

4.3.2.4 Products from *cyclooctene (S16)*

Cyclooctene oxide (P16c) has been identified by comparison with an authentic sample.
4-Cycloocten-1-one (P16f) and **2-cycloocten-1-one (P16b)**
The crude obtained collecting six photoreactions carried out on 65 μL (0.5 mmol) of substrate **S16** and 275 μL (1.5 mmol) of DTBP has been purified by flash chromatography on silica gel (eluent: hexane/ethyl acetate 1:1) leading to 16 mg of a colorless liquid (> 85% purity by GC) identified by ^{1}H NMR as a mixture 8 :1 of **4-cycloocten-1-one (P16f)** and **2-cycloocten-1-one (P16b)** in line with spectroscopic data reported in the literature.[S12,S13]
^{1}H NMR (700 MHz, $CDCl_3$): δ 5.79-5.71 (m, 2H), 2.56 – 2.48 (m, 6H), 2.21 (m, 2H), 1.66-1.62 (m, 2H).
3-Cycloocten-1-one (P16e) has been identified by GC-MS:
GC-MS (EI, 70 eV): m/z (%): 124 (12), 111 (7), 97 (22), 95 (20), 83 (29), 80 (41), 67 (83), 55 (100), 54 (55) , 41 (89), 39 (54).
2-(*tert*-butoxy)-cyclooctan-1-one (P16g) (or **2-cumyloxy-cyclooctan-1-one)** have been identified by GC-MS:

GC-MS (EI, 70 eV): m/z (%): 198 (2), 141 (14), 95 (25), 57 (100), 41 (29).
GC-MS (EI, 70 eV): m/z (%): 260 (2), 141 (100), 91 (31), 41 (15).

4.3.2.5 Products from *Norbornene (S17)*

Norbornene oxide (P17c) has been identified by comparison with an authentic sample.
3-(*tert*-Butoxy)-bicyclo[2.2.1]heptan-2-one and **3-(*tert*-butoxy)-bicyclo[2.2.1]heptan-2-ol (P17g)** have been identified by GC-MS:
GC-MS (EI, 70 eV): m/z (%): 182 (1), 134 (12), 120 (6), 107 (17), 95 (65), 79 (21), 68 (100), 67 (82), 55 (18), 41 (35), 39 (32), 32 (15).
GC-MS (EI, 70 eV): m/z (%): 184 (1), 182 (1), 154 (5), 121 (11), 97 (17), 81 (15), 79 (16), 67 (9), 57 (100), 41 (29), 32 (18).

4.3.2.6 Products from *Styrene (S18)*

Styrene oxide (P18c) and **benzaldehyde (P18h)** have been identified by comparison with authentic samples.
2-(*tert*-Butoxy)-1-phenylethan-1-one (P18g) has been identified by GC-MS:
GC-MS (EI, 70 eV): m/z (%): 192 (1), 162 (10), 119 (9), 105 (100), 91 (14), 77 (23), 57 (50), 41 (17), 32 (1).

5. NMR spectra

N-methylazepan-2,7-dione (P7α)

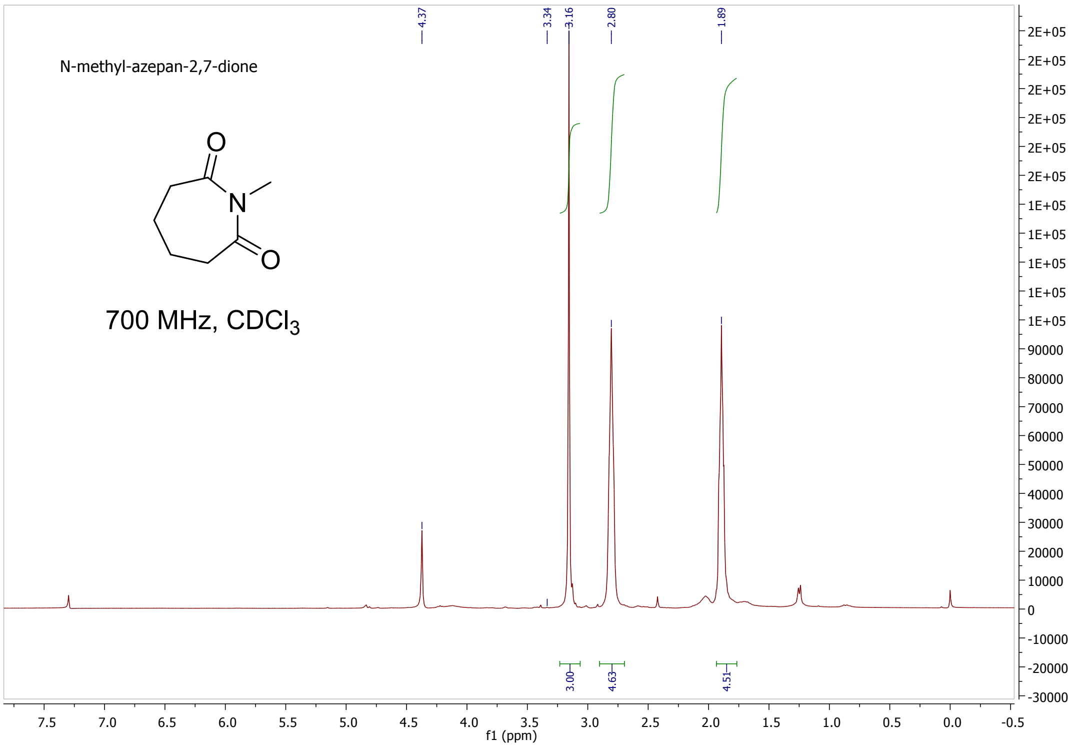

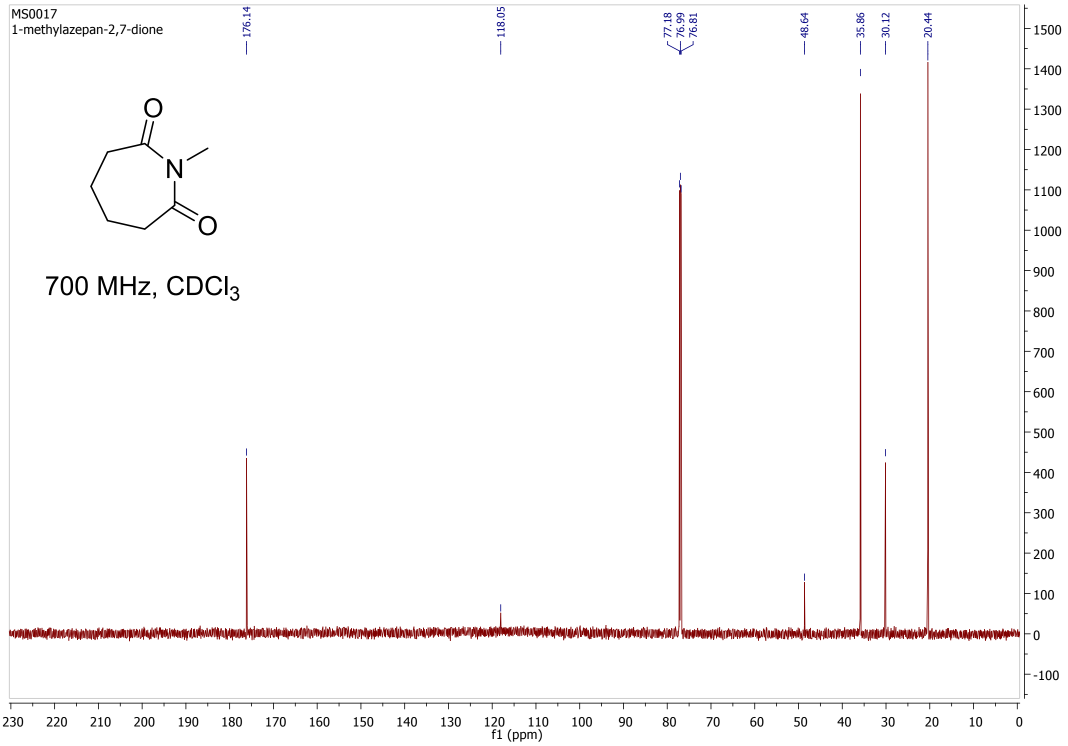

2-oxoazepan-1-carbaldehyde (P7*exo*)

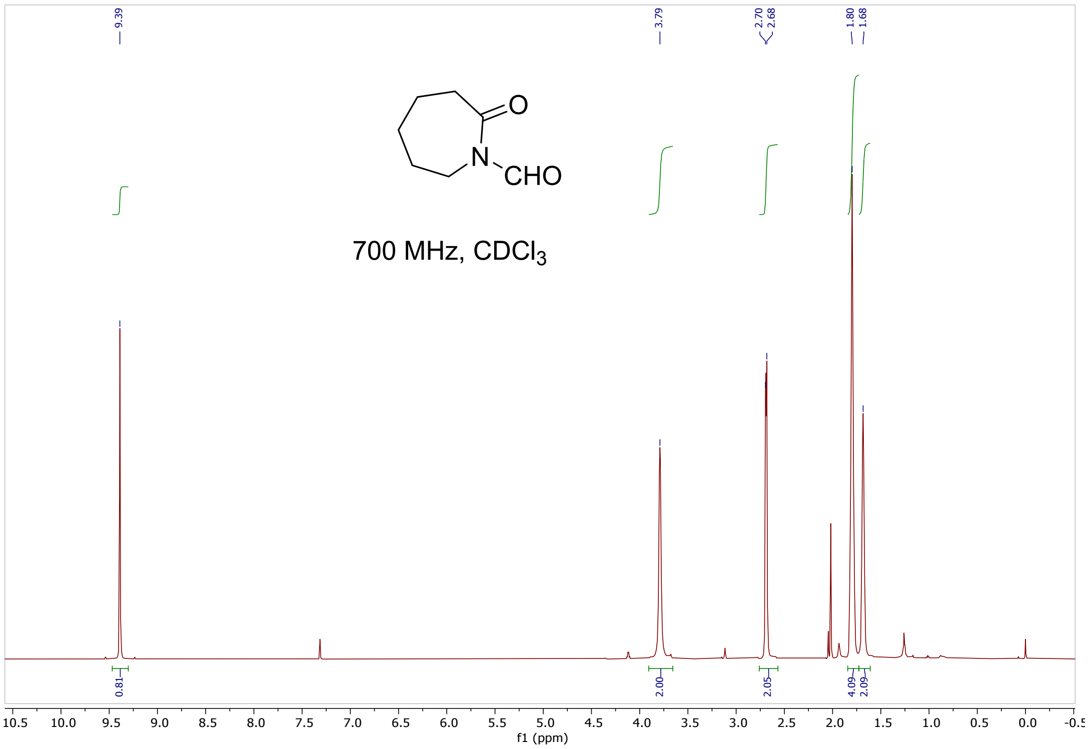

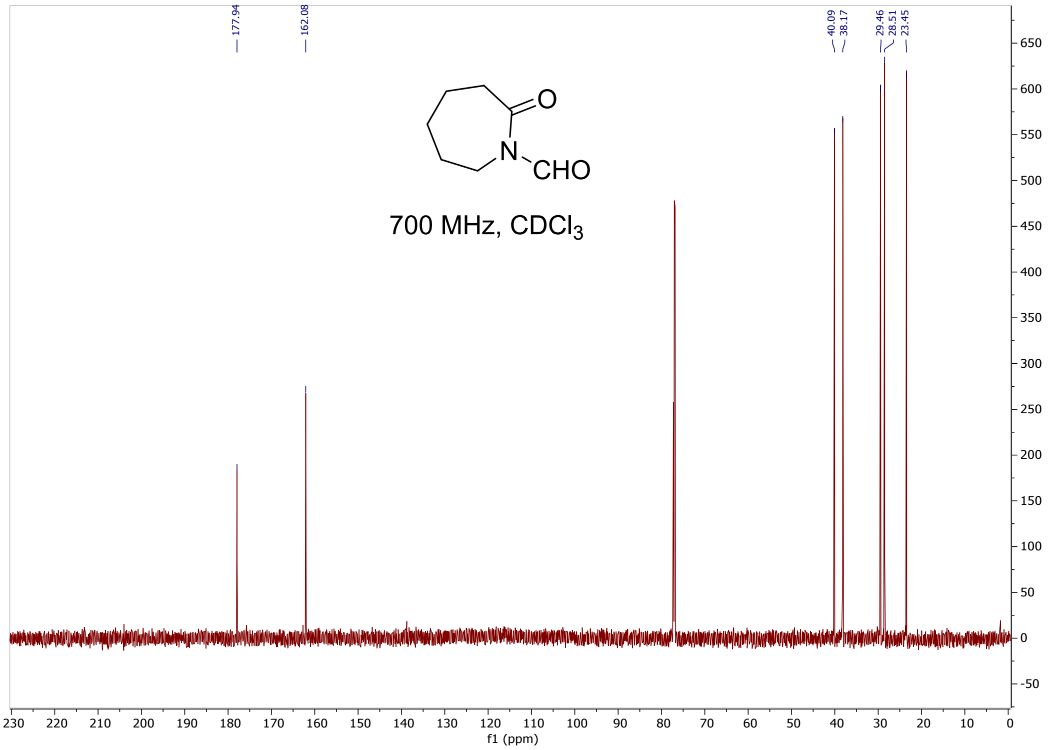

N-methylazocan-2,8-dione (P8α)

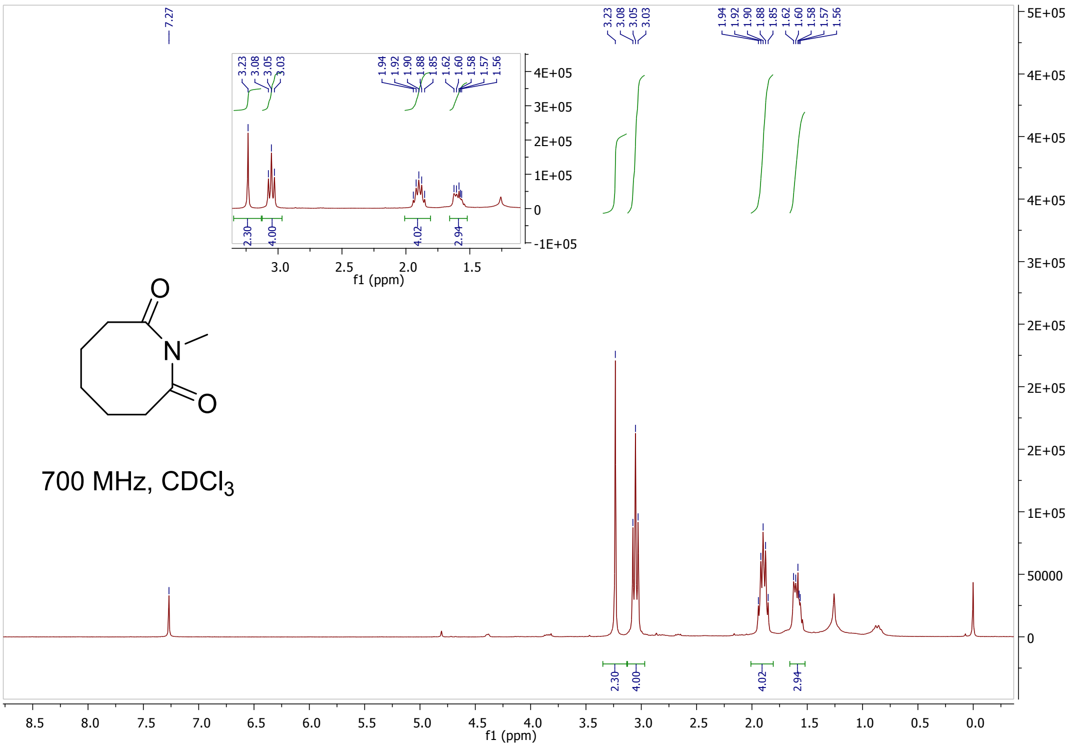

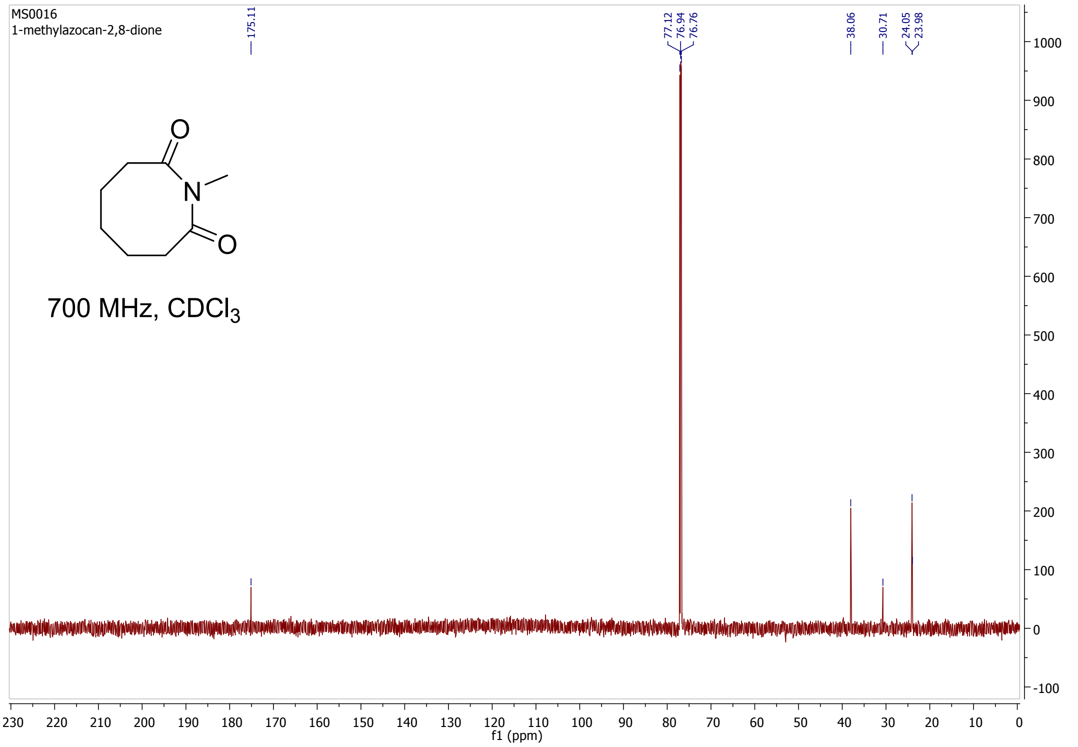

2-oxoazocan-1-carbaldehyde (P8*exo*)

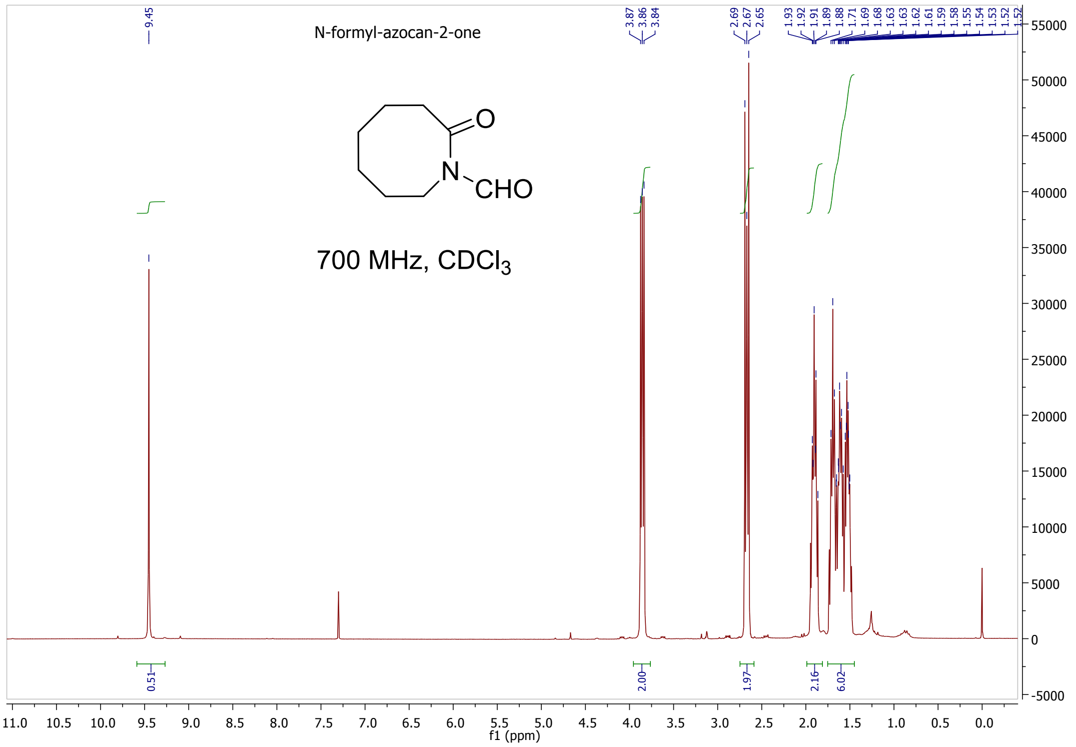

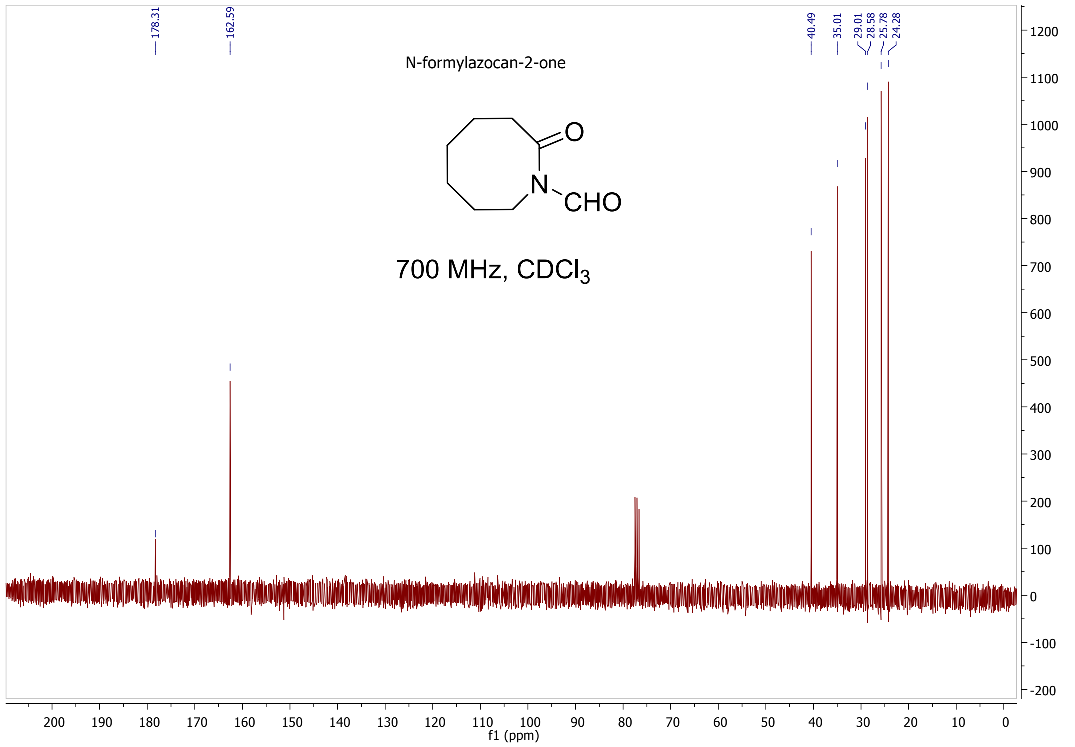

^{1}H NMR of the mixture containing *N*-methylazocan-2,7-dione (P8β)

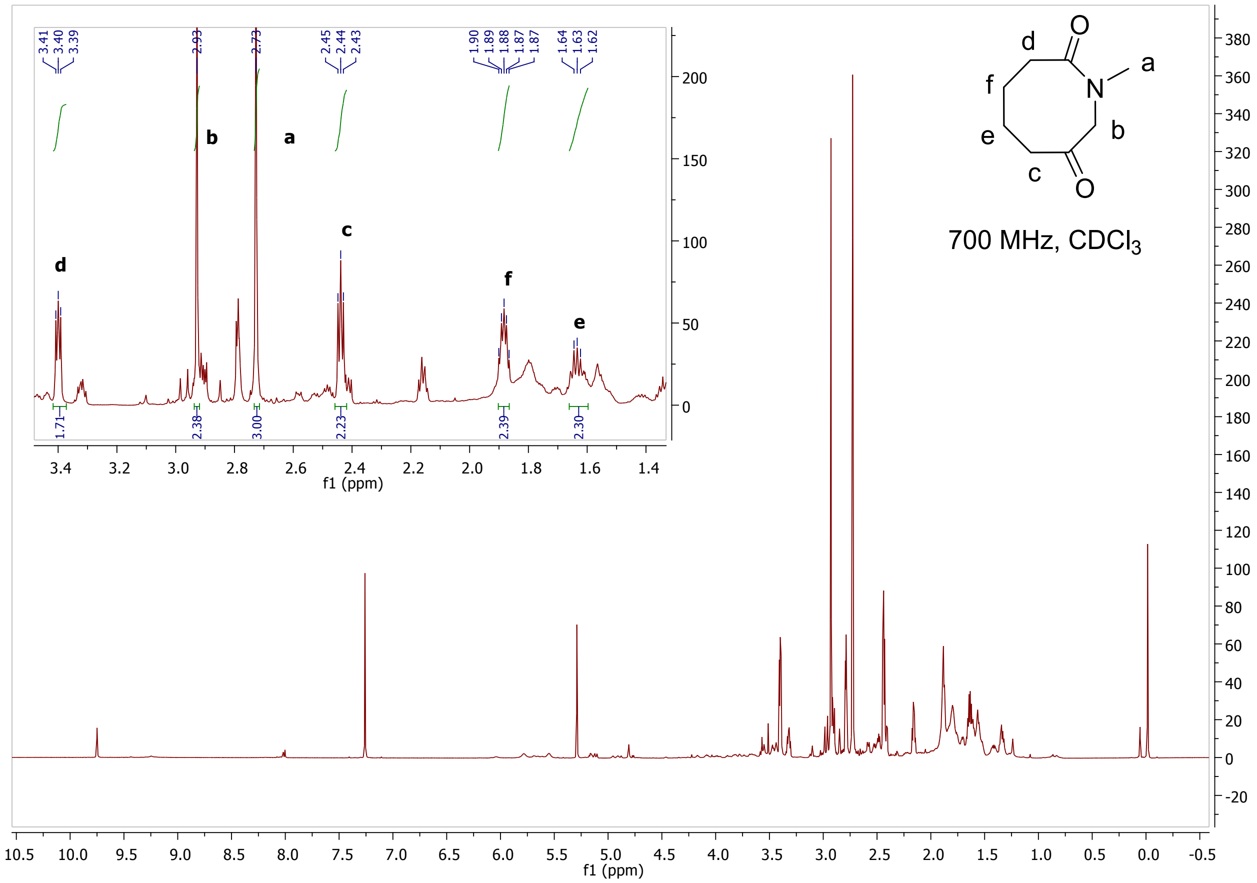

^{1}H NMR of the mixture containing *N*-methylazocan-2,6-dione (P8γ) and *N*-methylazocan-2,5-dione (P8δ)

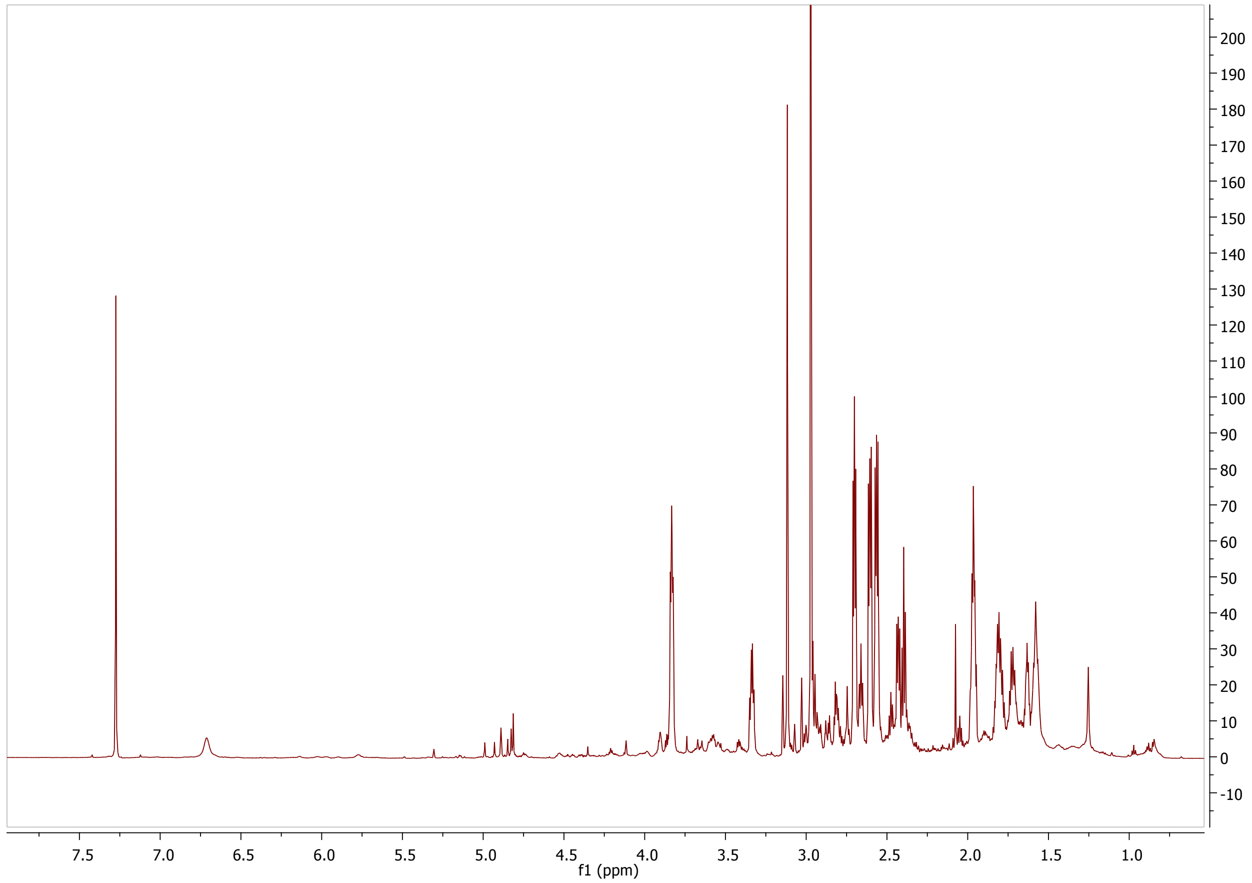

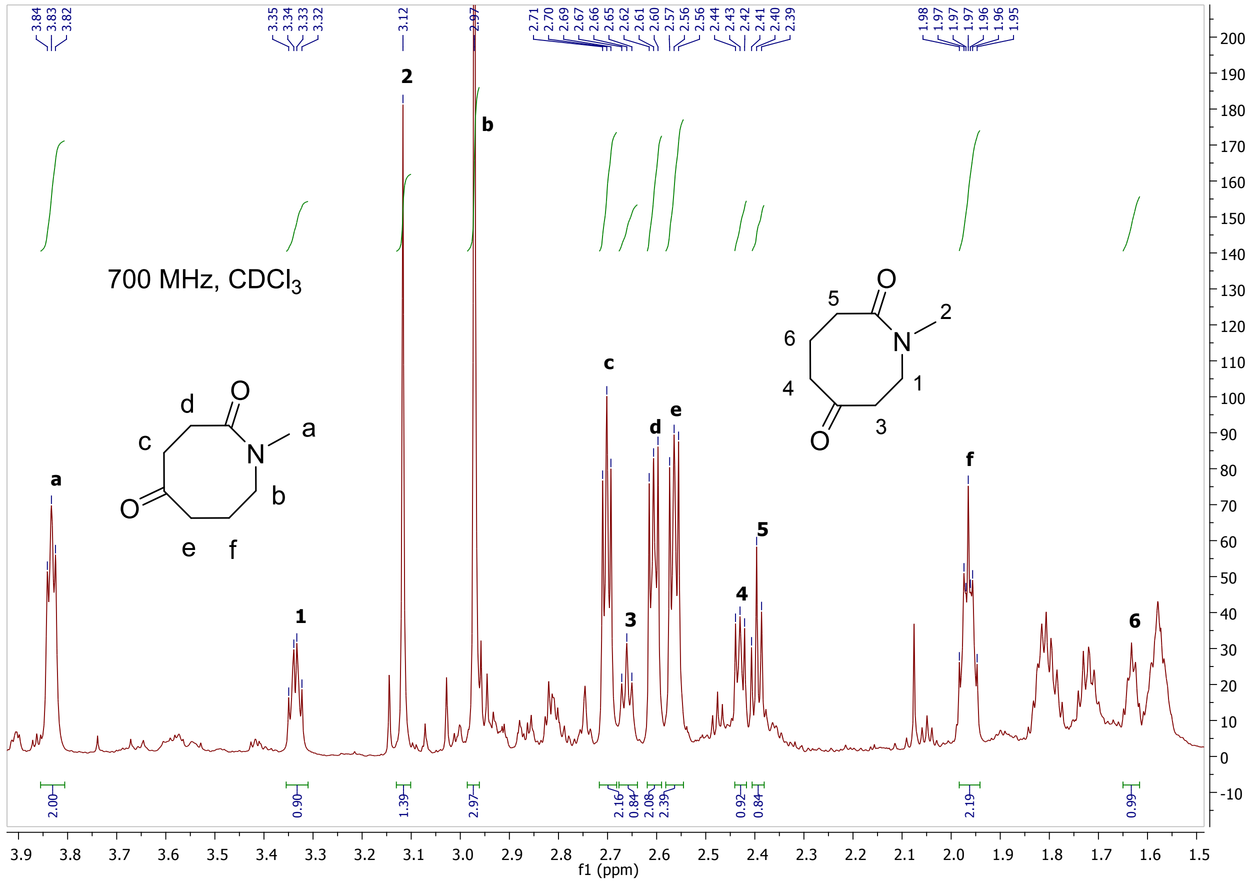

N-benzoylazepan-2-one (P11*exo*)

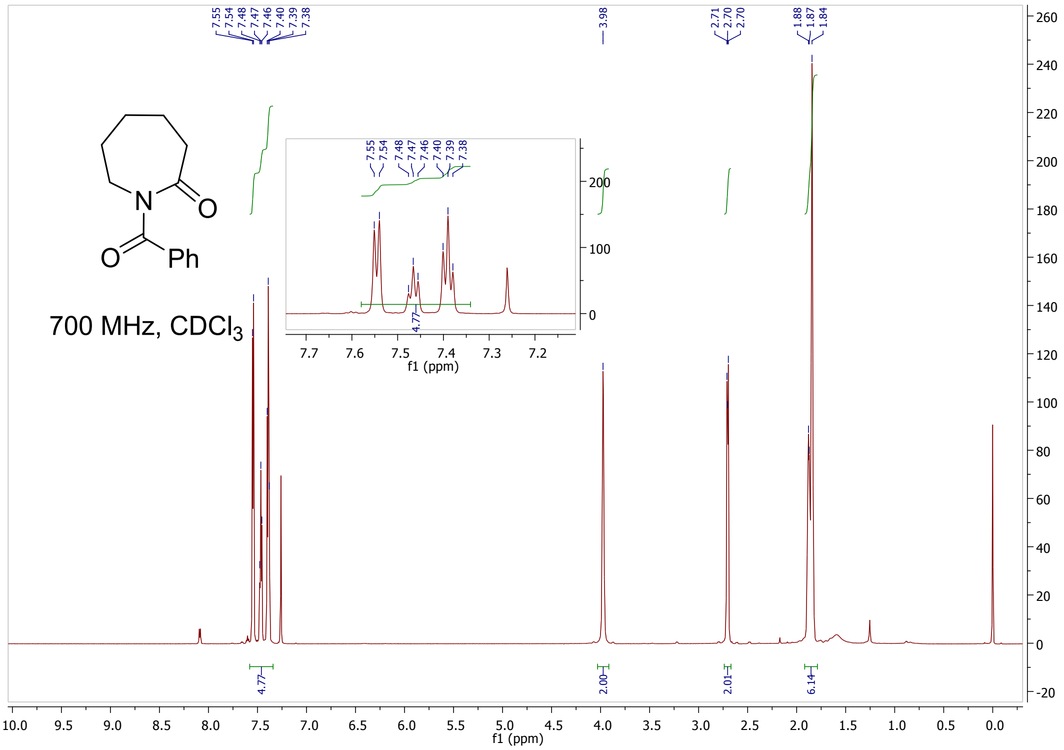

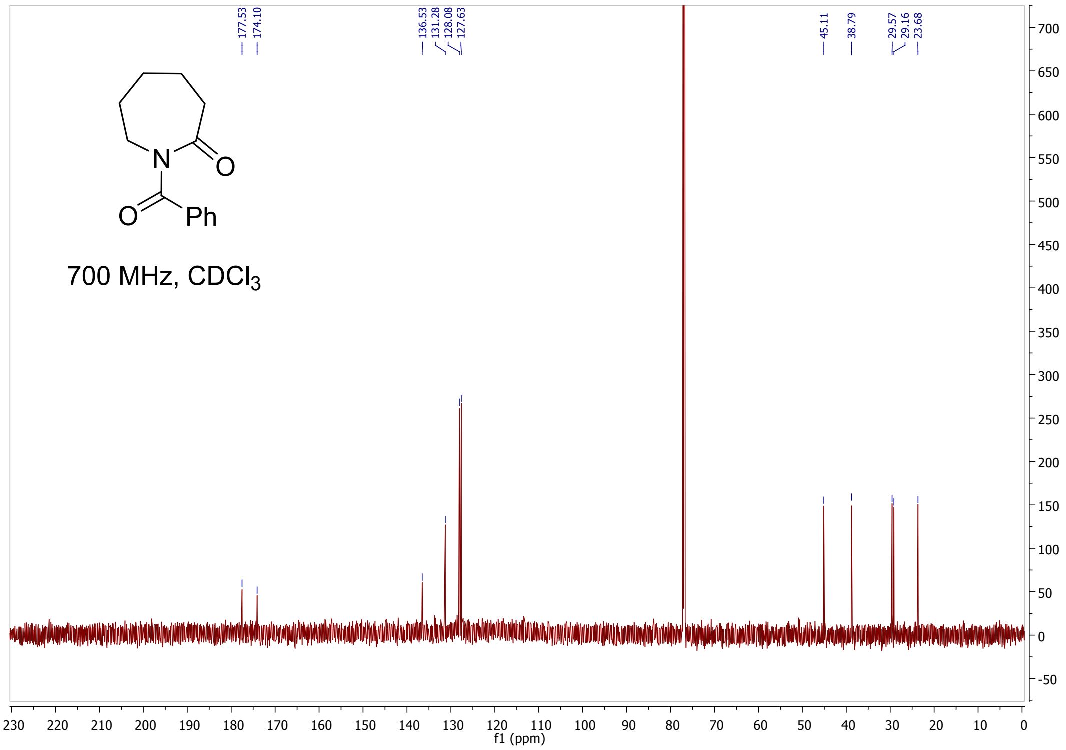

^{1}H NMR of the 1:1 mixture containing *N*-benzoylazepan-2-one (P11*exo*) and ***N*-benzylazepan-2,7-dione (P11α)**

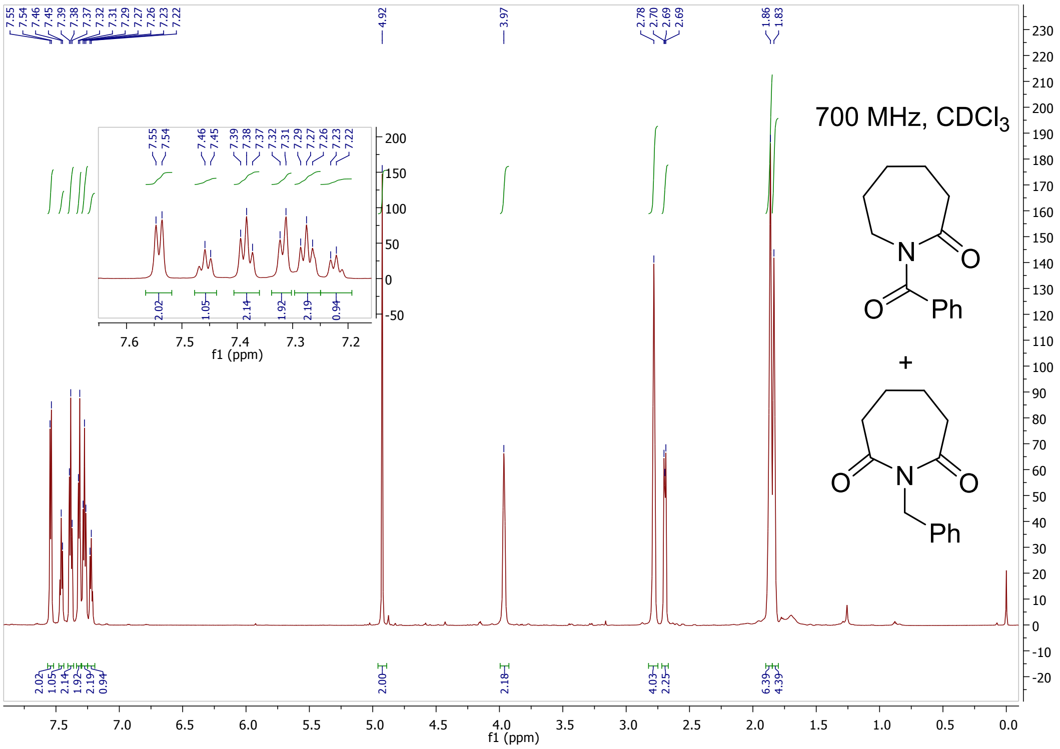

^{1}H NMR of the mixture containing *N*-benzoylazocan-2-one (P12*exo*) and *N*-benzylazocan-2,8-dione (P12α)

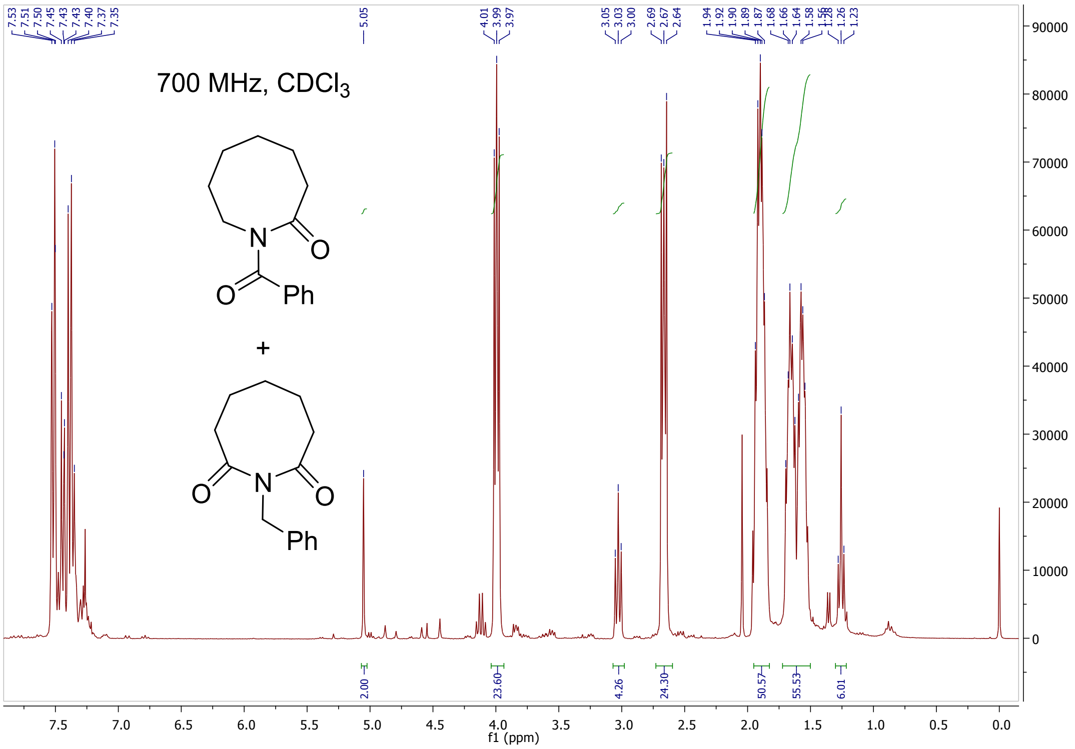

N-benzylazocan-2,7-dione (P12β)

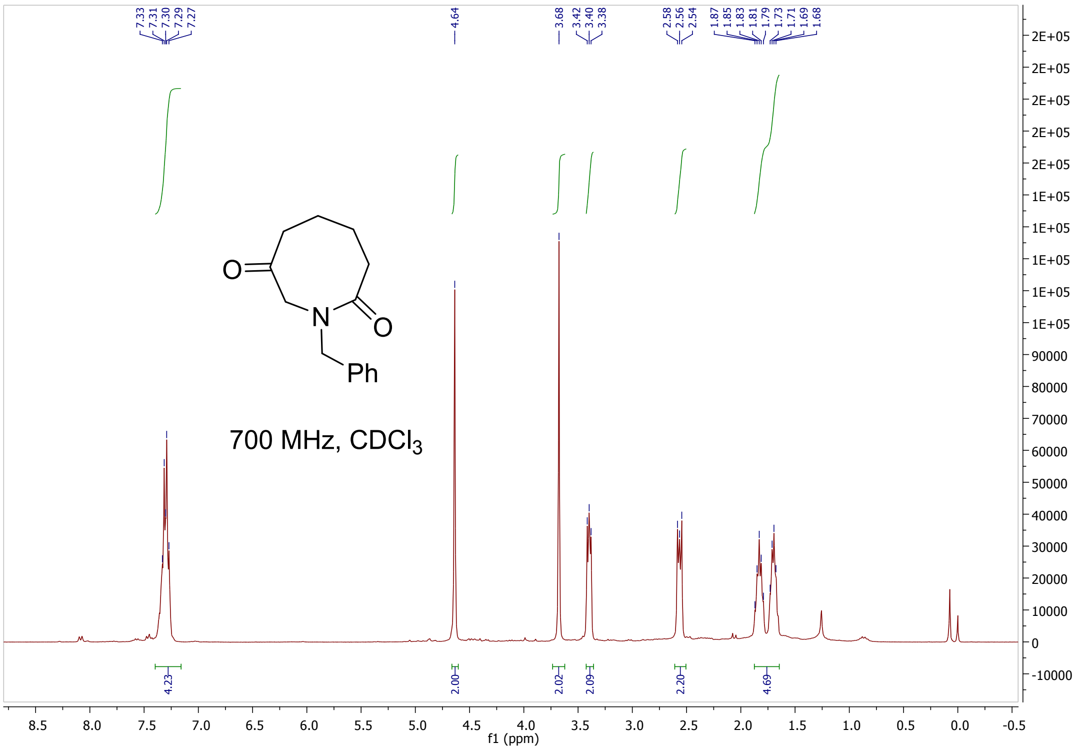

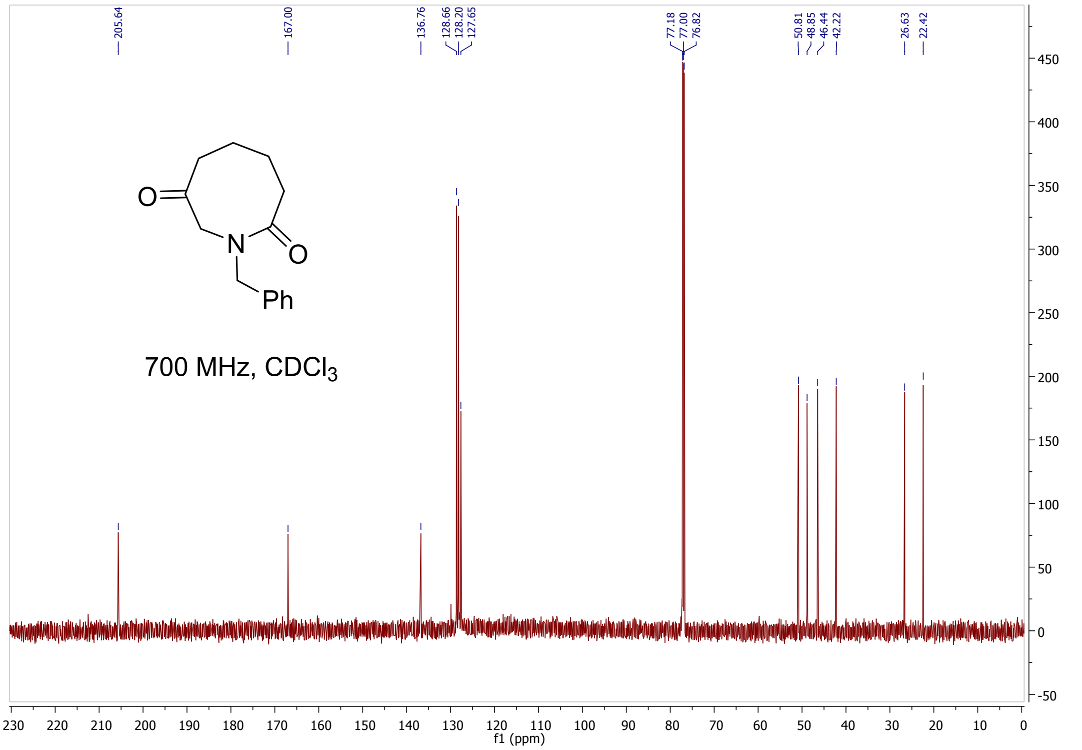

N-benzylazocan-2,6-dione (P12γ)

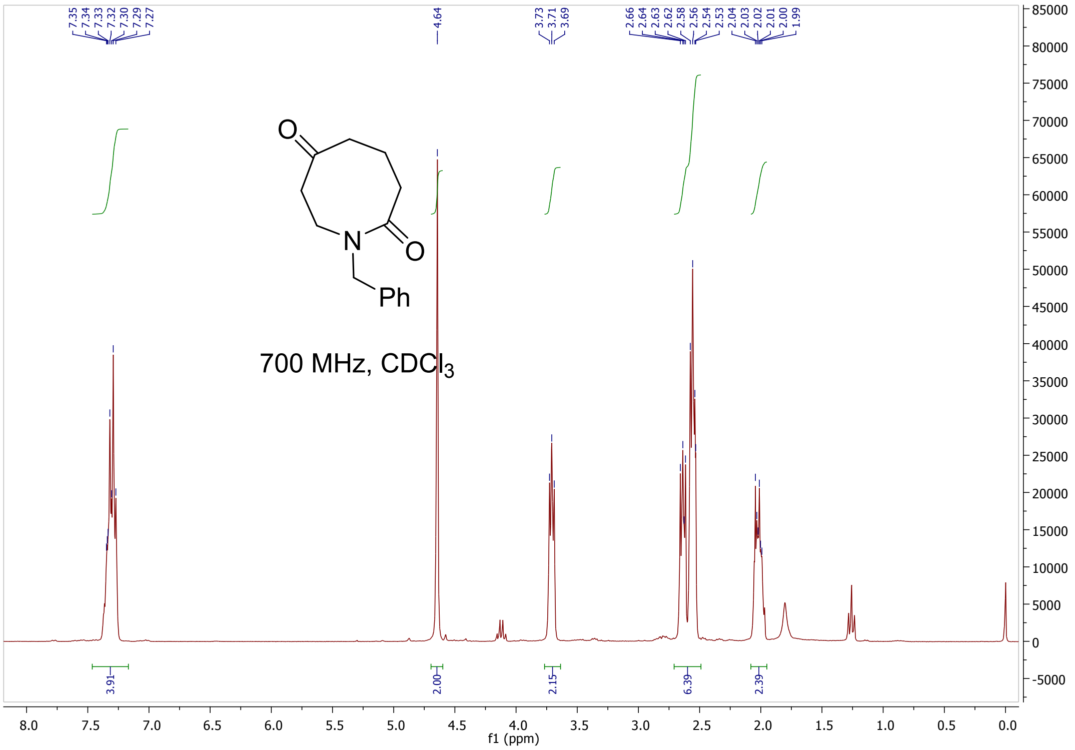

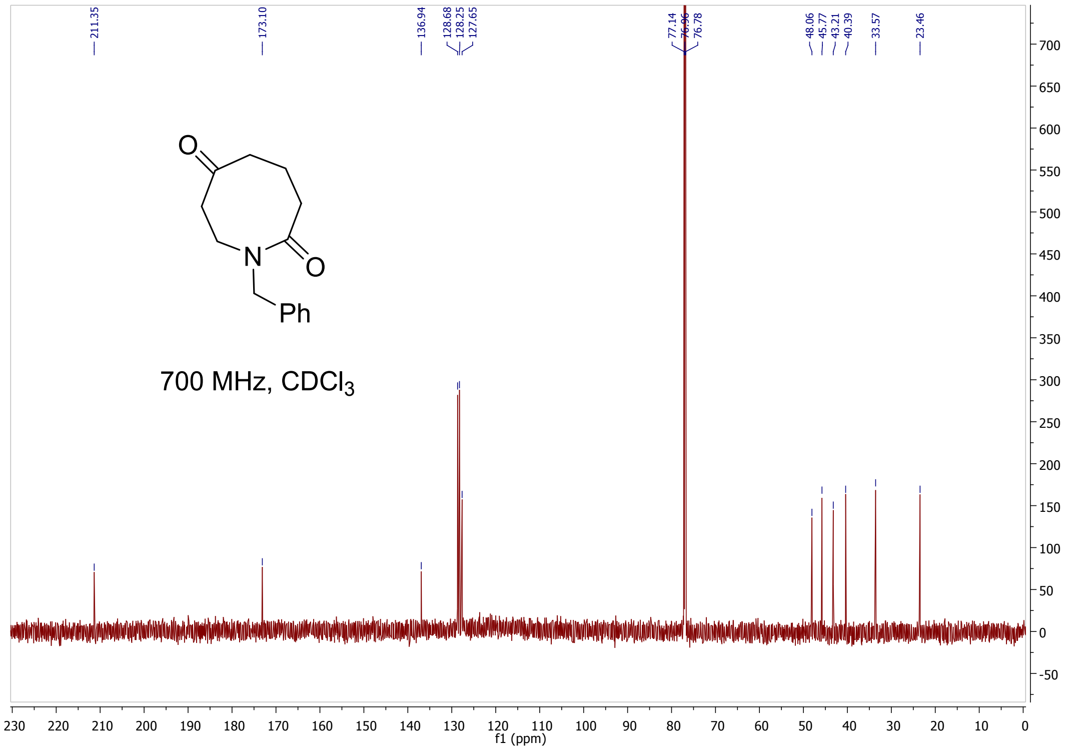

N-benzylazocan-2,5-dione (P12δ)

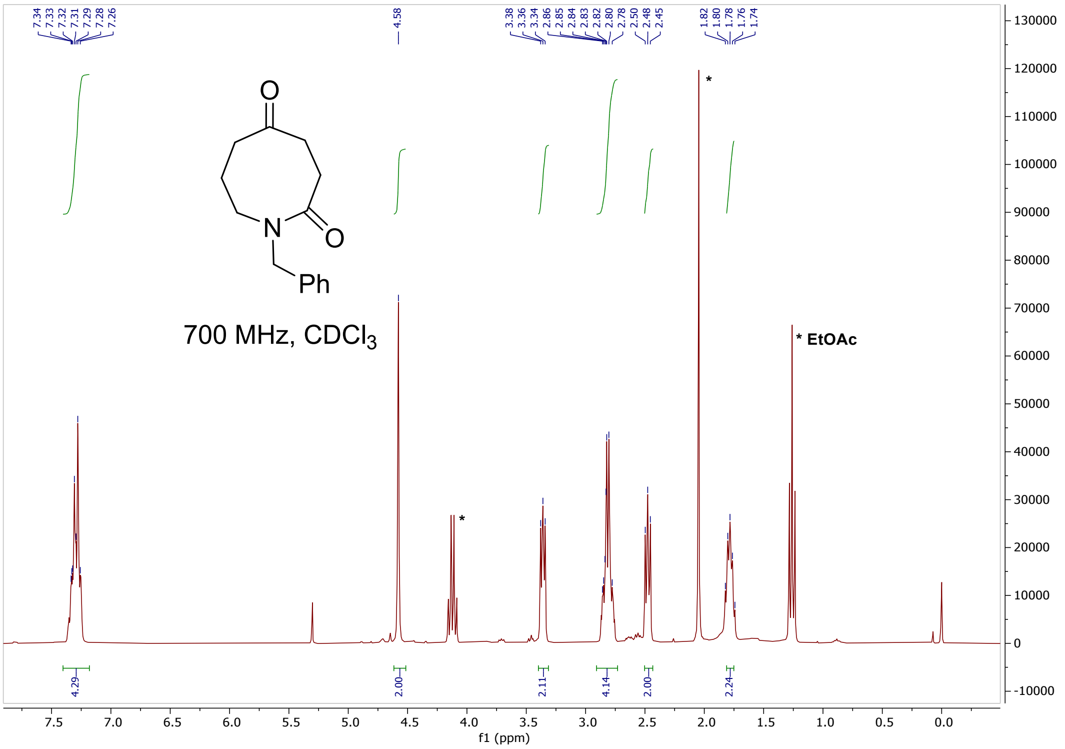

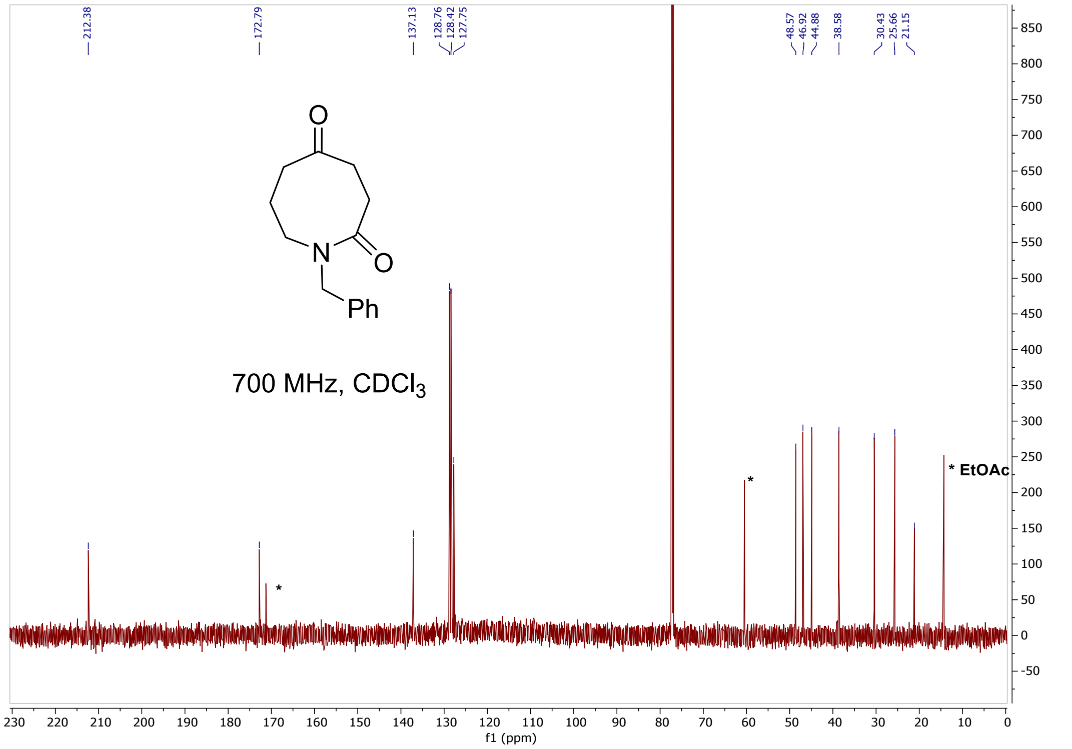

6. Computational studies

The optimized structures associated with the following BDE calculations are included in a zip file.

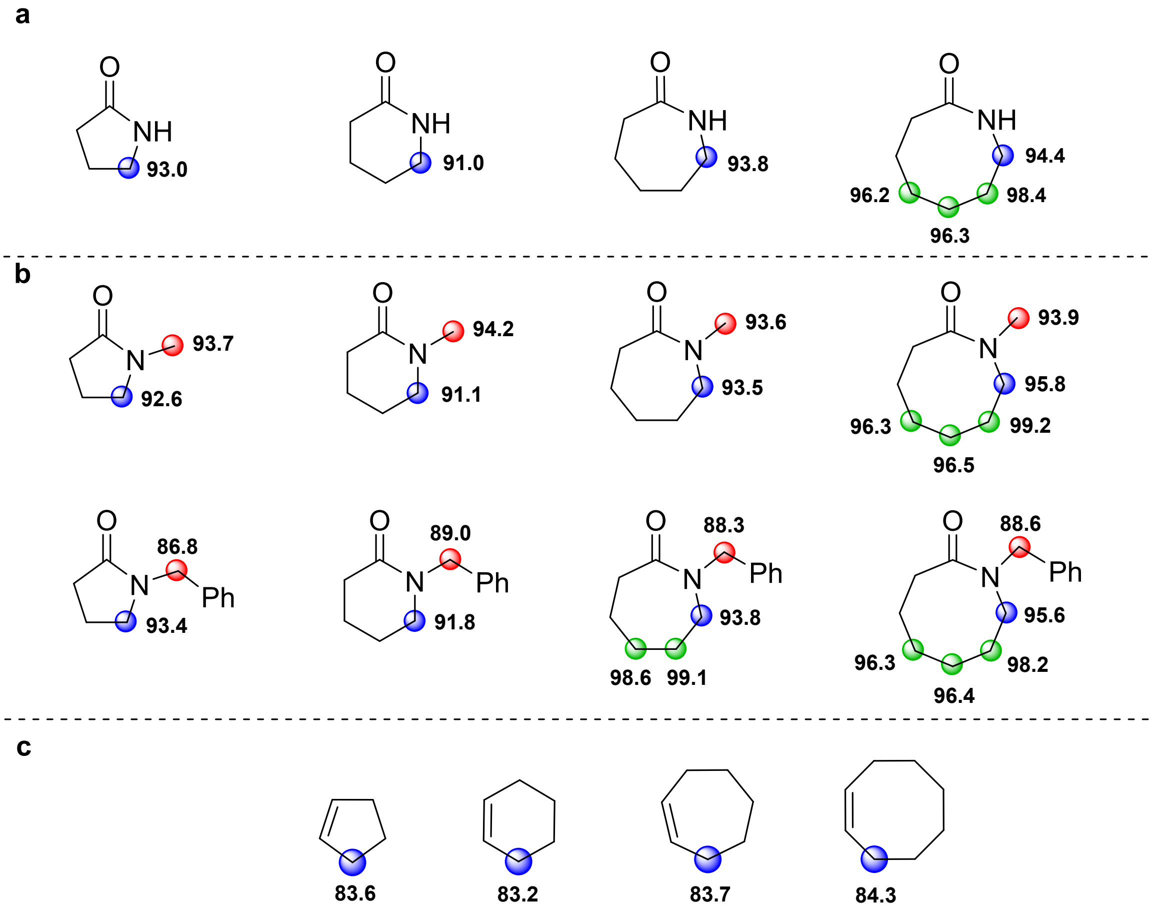

Figure S8. The bond dissociation energy (BDE) in kcal/mol for the lactams and alkenes calculated using the ωB97XD/pcseg-1-ACP approach. The optimized structures associated with the following BDE calculations are included in the accompanying zip file.

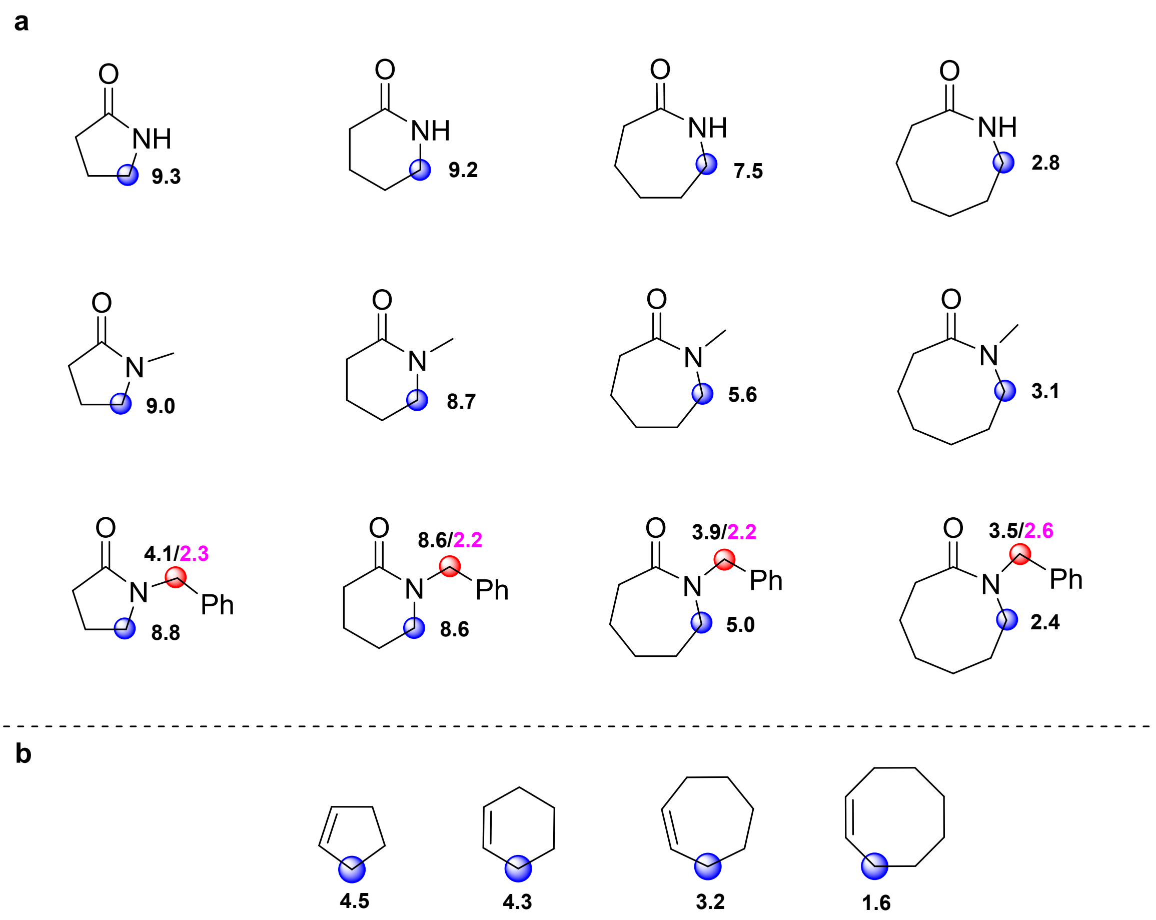

Figure S9. Secondary orbital interaction energies (kcal/mol) obtained from natural bond orbital (NBO) analyses. **a**) Black: Interaction energies involving C–H σ* bonds and nitrogen lone-pair orbitals at the positions indicated by the blue circles. Interaction energies involving benzylic C–H σ* bonds and nitrogen lone-pair orbitals at the positions indicated by the red circles. Pink: Interaction energies involving benzylic C–H σ* bonds and ring π-type orbitals at the positions indicated by the red circles. **b**) Interaction energies involving C–H σ* bonds and C–C π-orbitals.

7. References

S1. Moriarty, R. M. *J. Org. Chem.* 1964, **29**, 2748-2750.

S2. Fones, W.S. *J. Org. Chem.* 1949, **14**, 1099-1102.

S3. Gravel, M.; Thai, K.; Wang, L.; Dudding, T.; Bilodeau, F. *Org. Lett.* 2010*,* **12***,* 5708-5711.

S4. Hanada, S.; Tsutsumi, E.; Motoyama Y.; Nagashima H. *J. Am. Chem. Soc*. 2009, **131**, 15032-15040.

S5. Salamone, M.; Bietti, M. *Synlett* 2014, **25**, 1803-1816.

S6. H. Yin, L. Xu and N. A. Porter, *Chem. Rev.,* 2011, **111**, 5944-5972.

S7. (a) J. Hartung, N. Schneiders and T. Gottwald, *Tetrahedron Lett.*, 2007, **48**, 6027-6030. (b) T. Nakamura, W. K. Busfield, I. D. Jenkins, E. Rizzardo, S. H. Thang and S. Suyama, *J. Org. Chem.,* 1997, **62***,* 5578-5582. (c) P. C. Wong, D. Griller and J. C. Scaiano, *J. Am. Chem. Soc.,* 1982, **104***,* 5106-5108.

S8. Annese, C; D'Accolti, L.; Fusco, C.; Licini, G.; Zonta, C. *Chem. Eur. J.* 2017, **23**, 259-262.

S9. Yang, Y.; Wang, G.; Cao, X.; Yan, X.; Chen, L. *J. Chem. Res*. 2011, **35**, 657-658.

S10. Villa, M. V. J.; Targett, S. M.; Barnes, J. C.; Whittingham, W. G.; Marquez, R. *Org. Lett.*, 2007, **9**, 1631-1633.

S11. Suarez del Villar, I.; Gradillas, A.; Perez-Castells, J.; *Eur. J. Org. Chem.* 2010*,* 5850-5862.

S12. Clark, P. G.; Guidry, E. N.; Chan, W. Y.; Steinmetz,W. E.; Grubbs, R. H. *J. Am. Chem. Soc*. 2010, **132**, 3405–3412.

S13. Cui, L.; Liu, K.; Zhang, C. *Org. Biomol. Chem*. 2011, **9**, 2258-2265.
